# The interaction between the Nipah virus nucleocapsid protein and phosphoprotein regulates virus replication

**DOI:** 10.1038/s41598-018-34484-7

**Published:** 2018-10-30

**Authors:** Charlene Ranadheera, Roxanne Proulx, Mark Chaiyakul, Shane Jones, Allen Grolla, Anders Leung, John Rutherford, Darwyn Kobasa, Michael Carpenter, Markus Czub

**Affiliations:** 1 Department of Medical Microbiology and Infectious Diseases, University of Manitoba, Winnipeg, Manitoba Canada; 2 Zoonotic Diseases and Special Pathogens, National Microbiology Laboratory, Public Health Agency of Canada, Winnipeg, Manitoba Canada; 3 Blood Borne Pathogens and Hepatitis, National Microbiology Laboratory, Public Health Agency of Canada, Winnipeg, Canada; 4 Faculty of Veterinary Medicine, University of Calgary, Calgary, Alberta Canada

## Abstract

Continued outbreaks of Henipaviruses in South Asia and Australia cause severe and lethal disease in both humans and animals. Together, with evidence of human to human transmission for Nipah virus and the lack of preventative or therapeutic measures, its threat to cause a widespread outbreak and its potential for weaponization has increased. In this study we demonstrate how overexpression of the Nipah virus nucleocapsid protein regulates viral polymerase activity and viral RNA production. By overexpressing the Nipah virus nucleocapsid protein *in trans* viral transcription was inhibited; however, an increase in viral genome synthesis was observed. Together, the bias of polymerase activity towards genome production led to the severe inhibition of viral progeny. We identified two domains within the nucleocapsid protein, which were each independently capable of binding the viral phosphoprotein. Evident by our data, we propose that the nucleocapsid protein’s ability to interact with the phosphoprotein of the polymerase complex causes a change in polymerase activity and subsequent deficiency in viral replication. This study not only provides insights into the dynamics of Henipavirus RNA synthesis and replication, but also provides insight into potential targets for antiviral drug development.

## Introduction

Nipah virus (NiV) is a highly virulent zoonotic pathogen that can cause serious neurological and respiratory disease leading to death in both humans and animals^1–4^. It was first observed in Malaysia and Singapore in 1998 and is an emergent zoonotic disease in areas of South Asia with case fatality rates as high as 75%^1–6^. The first cases of human to human transmission were observed in Bangladesh during an outbreak in 2004, a property of infection that continues to manifest in subsequent outbreaks^7,8^. Severe disease and high mortality, human to human transmission, the lack of vaccines and antiviral therapies for human use, and increasing globalization have increased the fears of these viruses causing a widespread epidemic and its introduction into parts of the world where these viruses do not typically circulate.

NiV, along with Hendra virus (HeV), is a member of the genus Henipavirus within the family *Paramyxoviridae*. The NiV genome is 18.2 kb long, consisting of six viral genes encoding nine viral proteins. The inner structural component of the virion, the ribonucleoprotein (RNP) complex, is composed of the nucleocapsid protein (N), the phosphoprotein (P), the RNA-dependent RNA polymerase (L), and viral genomic RNA. The RNP complex is essential and sufficient for the synthesis of viral RNA^9^. The polymerase complex has been proposed to have two distinct functions, transcriptase and replicase activity^10–14^. The transcriptase, present in mature virions, is responsible for transcription of viral mRNAs upon entry into the cell and is composed of viral L and P proteins, and possible cellular factors^14–21^. The transcriptase is converted to a replicase and is responsible for the production of anti-genomic and genomic viral RNA and its concomitant encapsidation with N proteins^14,22,23^. The relative availability of the N protein is believed to be important for the activation of replicase activity, genome encapsidation and regulating viral RNA synthesis^24–26^. However, the mechanism(s) controlling these actions is not fully understood.

We established an experimental system that overexpressed NiV N in cells prior to NiV challenge. We show for the first time a substantial suppression of NiV transcription and translation, causing a subsequent decline in production of infectious virus when recombinant NiV N is present. We also visualized a shift from viral mRNA synthesis towards production of viral genome as recombinant NiV N protein increased, confirming the role of the N protein in regulating genome production. Furthermore, we identified two domains within NiV N, which are individually capable of binding NiV P and are associated with disrupting polymerase activity and subsequent viral replication. Our results suggest that recombinant NiV N present at the time of infection inhibits viral mRNA transcription and promotes viral genomic RNA synthesis mediated through a mistimed interaction with the NiV P, subsequently causing the overall inhibition of viral replication.

## Results

### Increased expression of NiV N causes reduced levels of viral transcriptase activity

**Figure 1:**
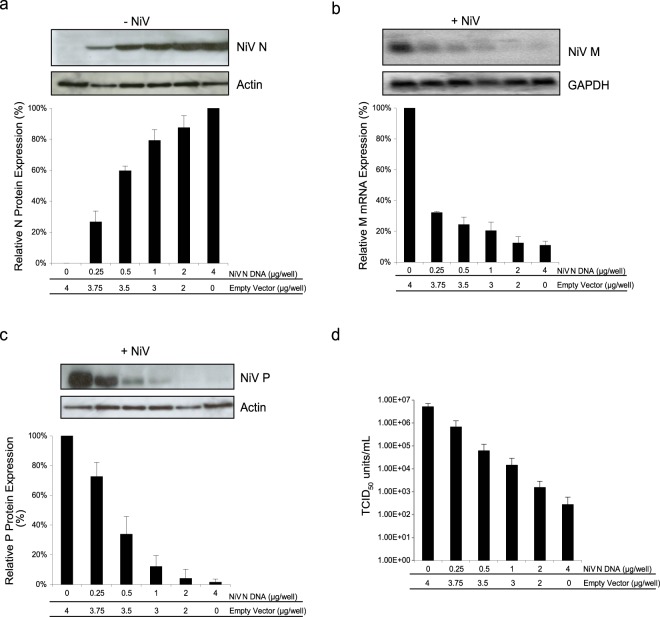
Effects of recombinant NiV N expression on NiV replication. Cells were transfected with increasing amounts of plasmid DNA encoding the NiV N gene. (**a**) 48 hours post transfection, cells were harvested and analyzed by western blot using a monoclonal antibody against NiV N. Western blots were quantified by densitometry and normalized to actin. Following transfection, a parallel set of cells were infected with NiV at an MOI of 1 for 24 hours. (**b**) RNA was extracted from NiV infected cells and analyzed by northern blot using a probe designed against the M gene. Northern blots were quantified by densitometry and normalized to GAPDH. (**c**) Total protein was harvested from NiV infected cells and analyzed by western blot for the expression of NiV P proteins. Western blots were quantified by densitometry and normalized to actin. (**d**) Supernatants were harvested, viral loads were determined by endpoint titration and TCID_50_/ml was calculated. All experiments were carried out in triplicate. Standard deviations of the mean were calculated. Blots have been cropped to ease visualization.

The nucleocapsid protein is known to play an integral role in viral replication by facilitating the functional transition of the viral polymerase from a viral transcriptase to a viral replicase with the concomitant encapsidation of full-length viral genomic RNA^14,22,23,25,27,28^. To gain specific understanding of the role of the N protein for NiV replication, 293 T cells were transfected with increasing amounts of a plasmid encoding NiV N, expression was monitored (Fig. 1a). A parallel set of cells were subsequently infected with NiV. Cells expressing high levels of NiV N revealed a 74% decrease in NiV matrix (M) mRNA in comparison to cells not expressing any recombinant NiV N (Fig. 1b). A negative correlation was observed between increasing N protein levels and viral M mRNA synthesis, while the steady state of cellular GAPDH mRNA was unaffected. A drop in viral mRNA correlated with a decrease in viral protein expression; western blot analysis confirmed the presence of recombinant NiV N at the time of infection resulted in a 98% reduction of NiV P expression (Fig. 1c). Together, it appears that viral transcription is impaired in the presence of increasing amounts of recombinant NiV N protein.

### Increasing expression of recombinant NiV N protein correlated with a decline in *de novo* viral particle production

The overall effect of impairing viral transcription and translation was evaluated by quantifying progeny virus production by TCID_50_ assays. Cells transiently expressing recombinant NiV N produced 2.76 × 10^2^ infectious units, whereas cells that did not express any recombinant NiV N generated 5.17 × 10^6^ infectious units, demonstrating a decrease in viral titres by approximately 4 orders of magnitude (Fig. 1d). While reduction of viral transcription and translation was impaired by approximately 1 order of magnitude, the combined effect impacted the production of progeny viruses dramatically. We observed similar findings when these experiments were replicated in BHK cells, demonstrating a cell line independent phenomenon (Supplemental Fig. S1). Overall, the expression of recombinant NiV N at the time of infection caused a negative effect on subsequent viral transcription, viral translation, and *de novo* virion production.

### Expression of recombinant NiV P does not modify the expression levels of NiV gene products

**Figure 2:**
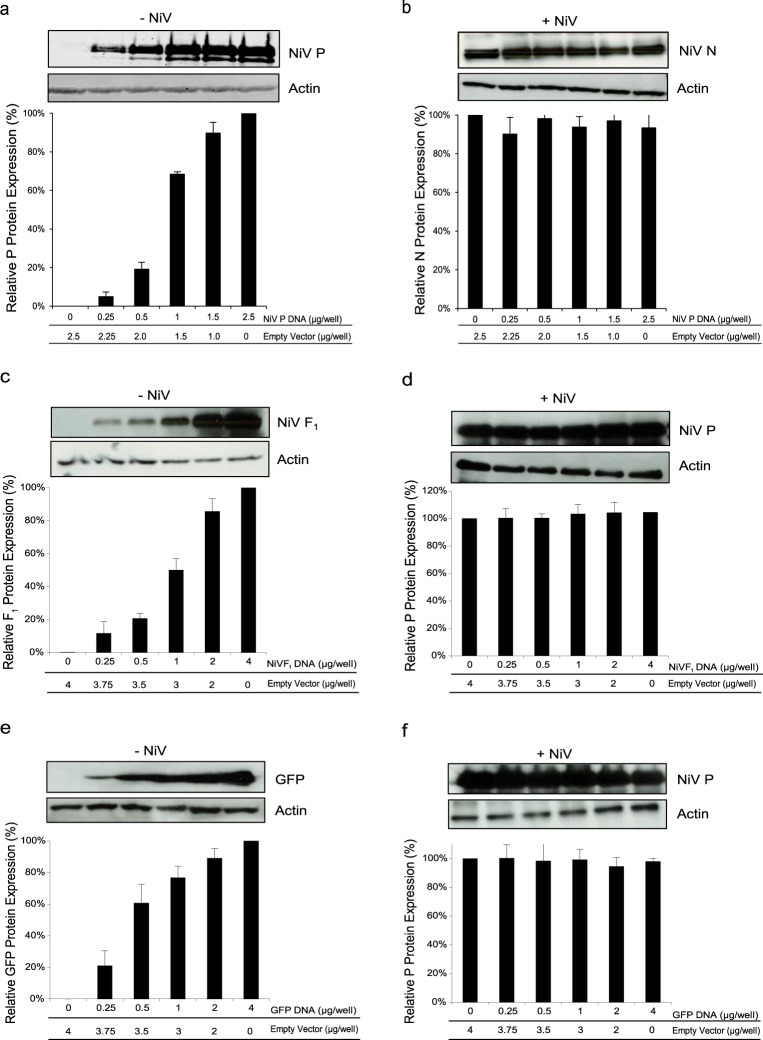
Effects of increasing expression of recombinant proteins on viral translation. Cells were transfected with increasing amounts of plasmid DNA encoding the (**a**) NiV P gene, (**c**) NiV F gene, or (**e**) GFP gene. 48 hours post transfection, cells were harvested and analyzed by western blot. Protein expression was quantified by densitometry and normalized to actin. Following transfection, a parallel set of cells were infected with NiV at an MOI of 1 for 24 hours. Total protein was harvested from NiV infected cells and analyzed by western blot for the expression of (**b**) NiV N proteins or (**d**) and (**f**) NiV P proteins. Western blots were quantified by densitometry and normalized to actin. All experiments were performed in triplicate and standard deviations of the mean were calculated. Blots have been cropped to ease visualization.

To assess specificity and ensure NiV N expression was responsible for the interruption of viral transcription and the subsequent production of infectious virus, we tested whether expression of other recombinant NiV proteins would also decrease the levels of NiV protein production. Cells expressing varying amounts of NiV P were infected with NiV. When viral protein expression was assessed during infection, we observed that expression of NiV N was found at equivalent levels regardless of the degree of recombinant NiV P being expressed (Fig. 2a and b). Similar results were also observed with other NiV proteins, such as the F protein (Fig. 2c and d), or the non-viral protein green fluorescence protein (GFP) (Fig. 2e and f).

### The presence of NiV N transcripts does not alter the expression of NiV proteins

**Figure 3:**
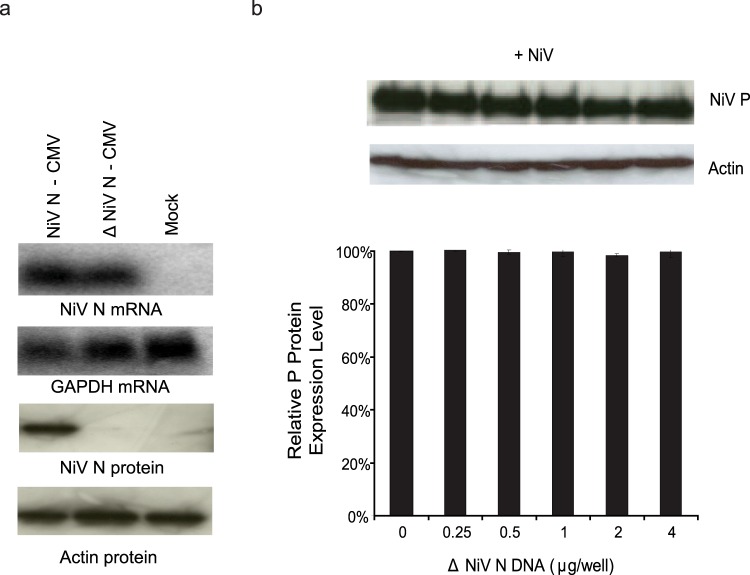
Effects of increasing NiV N mRNA on viral translation. (**a**) The translational start-sites of the NiV N gene were mutated to create an expression plasmid which produced NiV N transcripts but did not express NiV N proteins. 293 T cells were transfected with a NiV N competent expression plasmid (NiV N-CMV) and a NiV N incompetent expression plasmid (∆NiV N-CMV) for 48 hours. RNA was extracted and a northern blot was performed to visualize the presence of NiV N mRNA. Total cell protein was harvested and a western blot preformed to visualize the presence of NiV N proteins. (**b**) Following transfections, a parallel set of cells were infected with NiV at an MOI of 1 and total cell protein was harvested 24 hours later. A western blot was performed to analyze the expression of NiV P proteins. Western blots were quantified by densitometry and normalized to actin. All experiments were carried out in triplicate and standard deviations of the mean were calculated. Blots have been cropped to ease visualization.

To ensure that the NiV N mRNA being produced from the expression plasmid did not negatively impact viral replication, the translational start site of the NiV N gene was mutated to prevent NiV N proteins from being produced, while still maintaining the generation of NiV N mRNA (Fig. 3a). Cells expressing increasing amounts of NiV N mRNA but not NiV N proteins were infected with NiV. Viral translation was assessed and there did not appear to be any change in the expression of NiV P proteins when NiV N mRNA increased (Fig. 3b), indicating that NiV N mRNA did not silence viral replication.

### Increased expression of NiV N does not affect cell viability or cellular functions

**Figure 4:**
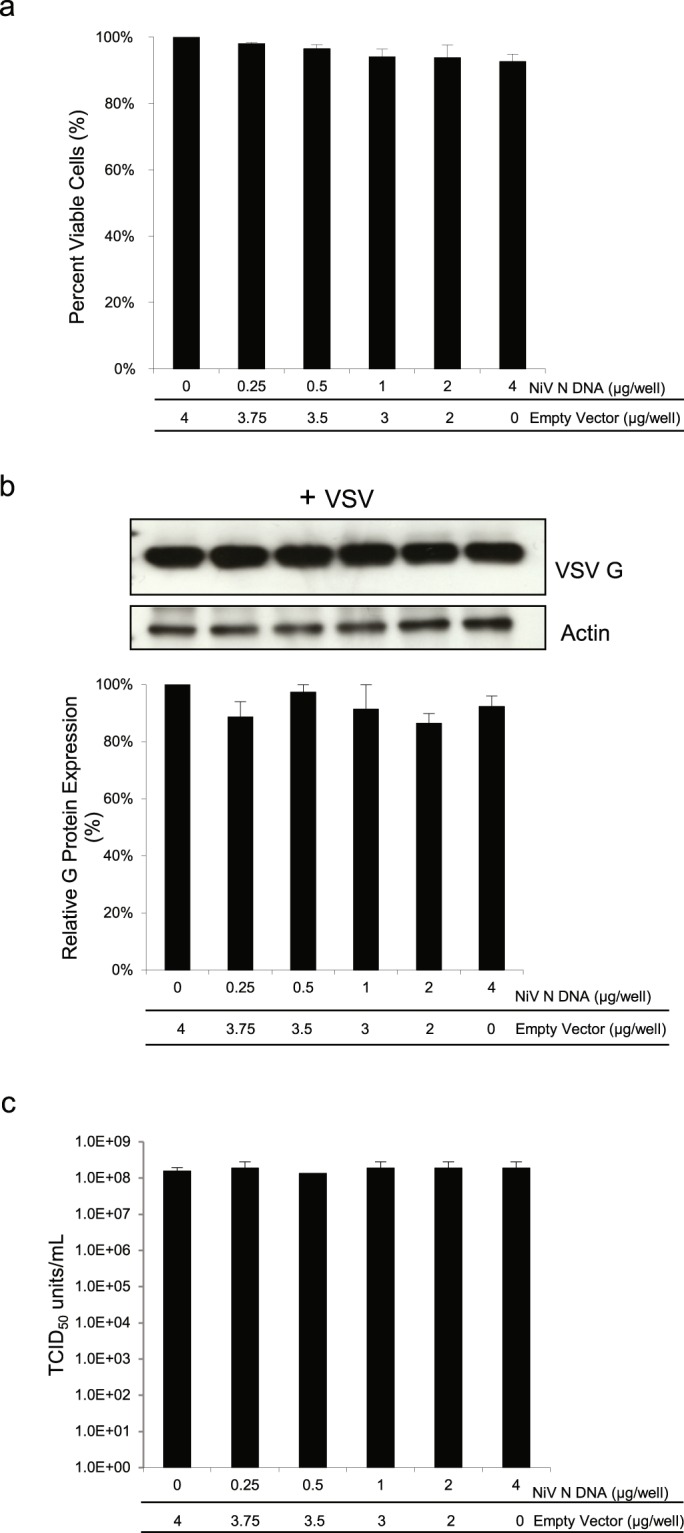
Effects of increasing expression of NiV N on cell viability and VSV replication. Cells were transfected with increasing amounts of plasmid DNA encoding the NiV N gene for 48 hours. Following transfection, (**a**) an XTT assay was performed to assess cell viability and a parallel set of cells were infected with VSV at an MOI of 1 for 24 hours. (**b**) Total cell protein was harvested to quantitate expression of VSV G by western blot and (**c**) supernatants were harvested for titration of viral loads. Western blots were quantified by densitometry and normalized to actin. All experiments were done in triplicate and standard deviations of the mean were calculated. Blots have been cropped to ease visualization.

Another explanation for the impairment of viral transcription could be that the expression of recombinant NiV N creates a general cytotoxic environment for the cell by inhibiting normal cellular functions such as protein translation or that it activates the cells antiviral responses, such as production of interferon causing impairment to viral replication. An XTT assay was employed to analyze mitochondrial function and viability of cells expressing recombinant NiV N. The presence of NiV N (Fig. 1a) did not appear to have any significant effects on cell viability (Fig. 4a). To determine whether the expression of recombinant NiV N initiated any negative effects on cellular functions that prevented viruses from replicating, cells expressing recombinant NiV N were infected with VSV. Cells expressing varying amounts of NiV N protein were infected with VSV, and viral translation and production of progeny virus was assessed. VSV G expression remained the same regardless of NiV N expression (Fig. 4b). Likewise, there was no change in the production of infectious VSV particles when recombinant NiV N was expressed (Fig. 4c), confirming the specificity of our findings towards Henipavirus replication and that cellular functions were still able to support replication of other viruses.

### Inhibition of viral replication is not caused by the formation of NiV N aggregates or nucleocapsid-like structures

**Figure 5:**
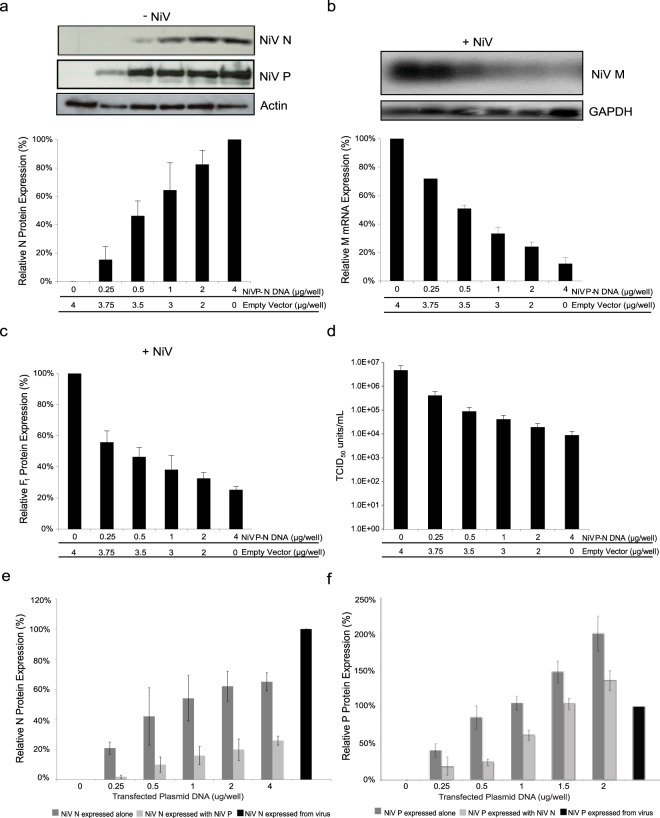
Effects of increasing recombinant NiV N proteins co-expressed with NiV P proteins on NiV replication. Cells were transfected with increasing amounts of plasmid DNA, containing both the NiV N gene and NiV P gene, which are driven by individual promoters encoded on one construct for 48 hours. (**a**) Cells were harvested and analyzed by western blot using a monoclonal antibody against NiV N. Following transfection, a parallel set of cells were infected with NiV at an MOI of 1 for 24 hours. (**b**) RNA was extracted from NiV infected cells and analyzed by northern blot using a probe designed against the M gene. Northern blots were quantified by densitometry and normalized to GAPDH. (**c**) Total protein was harvested from NiV infected cells and analyzed by western blot for the expression of NiV F_0_ proteins. (**d**) Supernatants were harvested, viral loads were determined by endpoint titration, and TCID_50_/ml was calculated. (**e**) Cells were transfected with increasing amounts of plasmid DNA expressing NiV N and/or NiV P protein for 48 hours or infected with NiV at an MOI of 1 for 24 hours. Total protein was harvested and a western blot was performed to visualize the presence of NiV N proteins or (**f**) NiV P proteins. All western blots were quantified by densitometry and normalized to actin. All experiments were carried out in triplicate and standard deviations of the mean were calculated. Blots have been cropped to ease visualization.

Typically, N proteins aggregate, and when expressed alone, form nucleocapsid-like structures by binding cellular RNA^27,29–33^. The presence of these structures could bias the incoming polymerase complexes away from true sites of replication and act as a sink for the viral polymerase, impairing viral transcription and production of progeny virus. The recognition capacity of P proteins for the viral template maintain N proteins in a soluble state until they are recruited to proper sites of viral replication^27,28,32–39^. To determine whether the presence of nucleocapsid-like structures were responsible for impairing viral replication, we confirmed the ability of the P protein to maintain NiV N in a soluble state (Supplemental Fig. S2). Upon confirmation, cells transiently expressing both NiV N and NiV P were infected with NiV. Similar to previous results, increasing amounts of recombinant NiV N in conjunction with recombinant NiV P (Fig. 5a), correlated with a dose-dependent decrease of viral mRNA (Fig. 5b), viral protein (Fig. 5c), and production of progeny virus (Fig. 5d). These results demonstrate that when NiV N is maintained in a soluble state, it is still capable of interfering with viral replication. The expression level of recombinant NiV N proteins, when expressed together with NiV P protein, was significantly lower than cells expressing NiV N proteins alone (Fig. 5e). When similar amounts of N protein were compared between the two systems (0.25 µg NiV N-CMV and 4 µg NiV P-NiV N-CMV) (Fig. 5e), NiV N expressed alone caused a reduction of progeny virus by 0.76 orders of magnitude (Fig. 1d), while cells expressing both NiV N and NiV P proteins demonstrated a decrease in viral titres by 2.5 orders of magnitude (Fig. 5d), indicating that the presence of NiV P proteins increased the capability of NiV N proteins to interfere with viral replication. In an attempt to further augment the inhibition of viral replication, we increased the expression of NiV P protein with constant expression of NiV N; however, no observable changes in reduction of viral replication was observed, suggesting the effect of NiV N is already at saturating levels in our experimental set up (Supplemental Fig. S3).

### Viral replicase activity is elevated with increasing expression of recombinant NiV N

**Figure 6:**
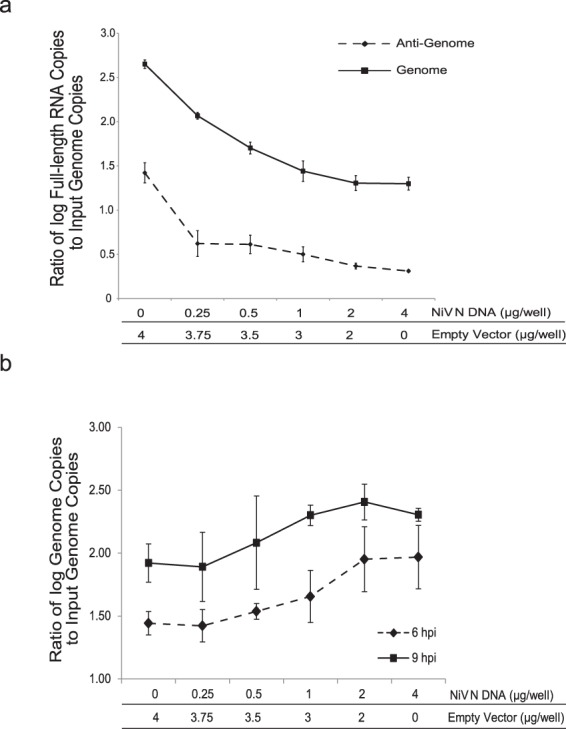
Effects of recombinant NiV N expression on input polymerase activity. Cells were transfected with increasing amounts of plasmid DNA encoding the NiV N gene. Total RNA was extracted from infected cells at (**a**) 24 hpi, (**b**) 0, 6 and 9 hpi, and 1 µg of RNA was analyzed by strand-specific RT and real-time PCR spanning the intergenic region between the NiV G and L genes. Each reaction was standardized to the input genome copy number after a 1 hour adsorption of virus and the ratio between genome copy numbers and input genome copy numbers was plotted. All experiments were carried out in triplicate. Standard deviations of the mean were calculated.

These experiments suggest that the impairment of a functional viral transcriptase is directly correlated with the expression of recombinant NiV N. In order to assess the replicase function of the viral polymerase, we examined the production of genomic and anti-genomic RNA using strand-specific reverse transcription coupled with real-time PCR. After infection, we observed a 22-fold and a 13-fold decrease in the copy numbers of viral genome and antigenome, respectively, when recombinant NiV N levels were high (Fig. 6a). Not surprisingly, the inhibition of viral transcription and translation inhibited the necessary gene products to propagate and visualize the production of *de novo* viral genomes. While we demonstrated that the transcriptase activity of the input viral polymerase was disrupted, we have yet to determine the functionality of the input polymerase. To address this question, a similar set of experiments were employed, except that RNA was harvested from cells at 0, 6 and 9 hours post-infection (hpi) to assess replicase activity of the input polymerase prior to subsequent rounds of viral replication. We observed an increase in viral genome production, as the amount of preexisting recombinant NiV N protein increased in cells (Fig. 6b). Increasing expression of recombinant NiV N seriously impaired transcriptase activity; however, the incoming viral polymerases had increased replicase activity, supporting the idea that the NiV N protein plays a role in transitioning the viral polymerase from viral transcription to genome production.

### NiV N contains two domains that are independently able to interact with NiV P

**Figure 7:**
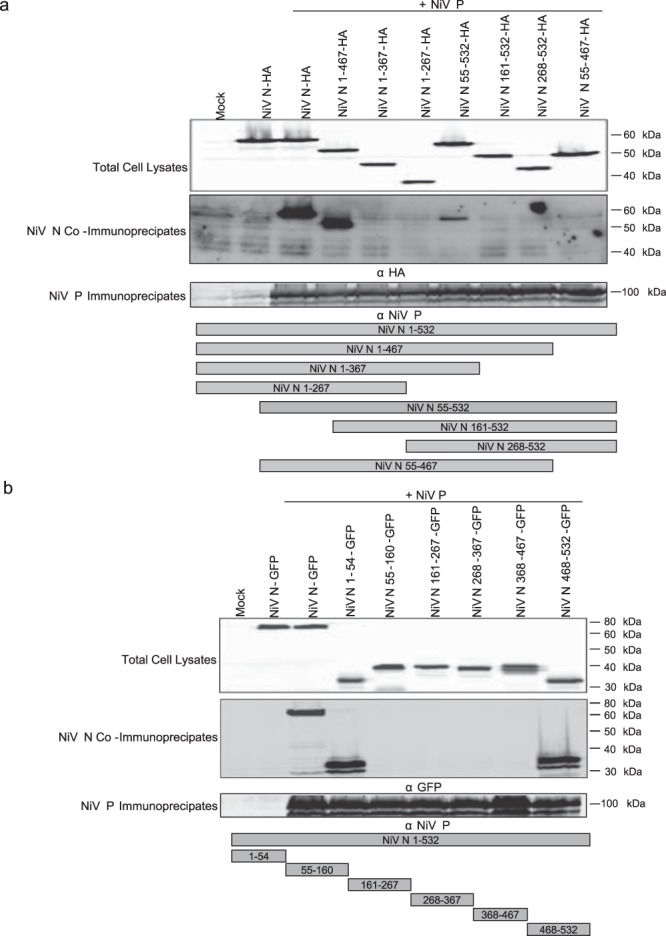
The ability of truncated NiV N proteins to interact with NiV P proteins. Cells were transfected with truncated NiV N constructs for 48 hours and protein expression was subsequently monitored from total cell lysates. A monoclonal antibody against NiV P was used to immunoprecipate NiV P proteins and co-immunoprecipate truncated NiV N proteins from cell lysates that transiently co-expressed (**a**) truncated NiV N proteins fused to an HA tag with NiV P proteins or (**b**) truncated NiV N proteins fused to GFP with NiV P proteins. Experiments were repeated at least three times. Blots have been cropped to ease visualization.

The interaction between N and P proteins is a vital process for viral RNA synthesis. The N protein is known to interact with the P protein to maintain itself in a useable state for viral replication^27,28,32–39^, and it also interacts with the P protein of the viral polymerase complex to mediate the production of viral antigenome and genome, and its encapsidation^14,23,25,37,40,41^. In an effort to better characterize how the NiV N protein regulates its viral polymerase activity, we focused on this interaction and determined if the region(s) necessary for binding NiV P were sufficient to cause a disruption to viral RNA synthesis. A series of systematic NiV N truncations were created and transiently co-expressed with NiV P. Transient expression of these constructs was confirmed by western blot prior to immunoprecipitation (Fig. 7a). Co-immunoprecipitation assays were conducted to assess the ability of the truncated proteins to interact with NiV P. Immunoprecipitation of NiV P effectively co-immunoprecipitated three NiV N-HA constructs: NiV N-HA, NiV N 1-467-HA, and NiV N 55-532-HA (Fig. 7a). The ability of NiV N 55-532-HA to interact with NiV P indicated that the N-terminal region of the protein is not required for binding; likewise, the interaction of NiV N 1-467-HA with NiV P suggests the C-terminal domain is also dispensable. However, when N was truncated from both the N- and C-terminal ends, NiV N 55-467-HA, binding of NiV P was abolished, instead suggested the possibility of two independent NiV P binding domains, one at each end of the protein. Interestingly, other constructs containing these terminal domains, NiV N 1-367-HA, NiV N 1-267-HA, NiV N 268-532-HA and NiV N 161-532-HA were not able to immunoprecipitate with NiV P. It is possible that these larger deletions had a negative effect on protein conformation and disrupted the natural folding patterns. To further identify the regions capable of binding NiV P, six non-overlapping fragments of the N protein were generated and fused to GFP. Prior to immunoprecipitation, transient expression of these constructs was confirmed by western blot (Fig. 7b). The immunoprecipitation of NiV P demonstrated interactions with three of the NiV N-GFP constructs: NiV N-GFP, NiV N 1-54-GFP, and NiV N 468-532-GFP (Fig. 7b). These results confirmed that two distinct regions on NiV N, aa1-54 and aa468-532, were both capable of interacting with NiV P. These findings confirmed the presence of a NiV P binding site on the C-terminal end of NiV N, as previously described^42^, and indicated the presence of a novel domain on the N-terminus of NiV N.

### The NiV P binding domains found on NiV N are capable of individually disabling viral replication

**Figure 8:**
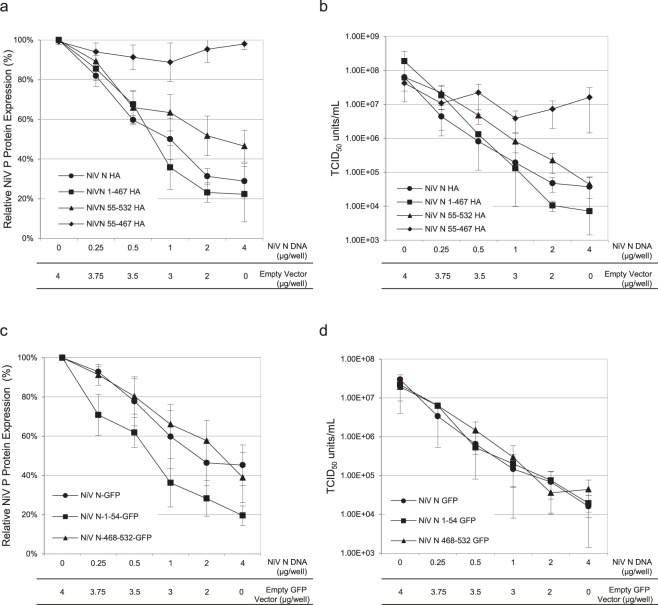
Analysis of NiV replication as expression levels of truncated NiV N-HA protein increase. All experiments were carried out in triplicate and standard deviations of the mean were calculated. Cells transiently expressing increased amounts of various truncated NiV N proteins were infected with NiV at an MOI of 1 for 24 hours. Lysates were harvested from cells expressing (**a**) NiV N truncations fused to an HA tag or (**c**) NiV N truncations fused to GFP and analyzed by western blots using a monoclonal antibody against NiV P. Western blots were quantified by densitometry and normalized against the expression of actin. Expression levels of viral NiV P in the absence of recombinant NiV N was set to 100% expression. Supernatants were harvested from cells expressing (**b**) NiV N truncations fused to an HA tag or (**d**) NiV N truncations fused to GFP, viral loads were determined by endpoint titration and TCID_50_/ml was calculated. All experiments were done in triplicate and standard deviations of the mean were calculated.

To define the importance of the interaction between NiV N and NiV P on viral replication, cells transiently expressing various truncated NiV N proteins were infected with NiV and the replication pathway was assessed as previously described. The first set of truncated proteins analyzed were: NiV N 1-467-HA, deficient in the C-terminal NiV P binding domain, NiV N 55-532-HA, deficient in the N-terminal NiV P binding domain, and NiV N 55-467-HA, deficient in both NiV P binding domains. Cells expressing recombinant NiV N 55-532-HA proteins demonstrated a 55% reduction in NiV P expression (Fig. 8a) and a 3.2 log_10_ decrease in infectious titres (Fig. 8b). Similarly, expression of NiV N 1-467-HA correlated with a decrease of viral NiV P by 79% (Fig. 8a) and a reduction of viral titres by 4.4 log_10_ (Fig. 8b). However, cells expressing NiV N 55-467-HA demonstrated no significant reduction in NiV P expression (Fig. 1a) or infectious virus production (Fig. 8b). Overall we observed a dysfunction of viral translation and virion production in the presence of increased amounts of recombinant NiV N-HA containing one or both NiV P binding domains. Notably, removal of both NiV P binding domains from NiV N abrogated its effects, suggesting that interaction between NiV N and NiV P is essential for inhibition of viral replication.

To confirm these results, we tested whether the NiV P binding domains alone, NiV N 1-54 or NiV N 468-532, could affect viral replication. Cells were transfected with truncated NiV N-GFP constructs and subsequently infected with NiV, as previously described. Western blot analysis and the titration of viral loads demonstrated that cells transiently expressing NiV N 1-54-GFP incurred an 80% reduction in NiV P expression (Fig. 8c) and a 3.1 log_10_ reduction in the production of progeny virus (Fig. 8d). Cells transiently expressing NiV N 468-532-GFP demonstrated a 61% reduction in NiV P expression (Fig. 8c) and a 2.7 log_10_ reduction in viral titre (Fig. 8d). Similarly, when co-expressed with NiV P, these constructs were also able to effectively inhibit viral replication (Supplemental Fig. 4). These results confirmed the influence that a peptide encoding one of the two NiV P binding sites of the NiV N had on abrogating viral gene expression.

## Discussion

We demonstrated that the overexpression of recombinant NiV N at the time of infection impaired viral transcription and translation, which subsequently caused a decline in viral genome synthesis and production of progeny virions. While the incoming viral polymerase lost transcription function, its replication competency increased evident by elevated levels of viral genome present at early time points, as the amount of recombinant NiV N protein increased in cells. We isolated the regions within the NiV N protein responsible for impairing viral replication, which coincided with regions capable of binding NiV P proteins. Two separate NiV P binding domains found on NiV N, corresponding to amino acids (aa) 1-54 and 468–532, were elucidated respectively. Our results demonstrated the presence of a novel NiV P binding domain found at the N-terminal end of NiV N and confirmed the findings of a previous study, which identified a NiV P binding domain within the C-terminal end of the protein^42^. A third domain suggested to bind NiV P was shown to reside between residues 135 and 146 of NiV N^43^; however, using our experimental set up, we were not able to confirm these findings.

Control experiments demonstrated that the impairment of NiV replication is dependent on the presence of recombinant NiV N, not NiV N transcripts or other recombinant proteins, and that cell viability and transcription/translation machinery are unaffected. The biological function of N proteins is to bind viral RNA; however, when expressed alone, it is known to aggregate and/or non-specifically bind cellular RNA forming cellular nucleocapsid-like structures and may inadvertently divert incoming polymerase complexes away from sites of replication^27,29–33^. The presence of P protein is believed to prevent non-specific binding of cellular RNA and recruit N proteins toward virus replication^27,28,32–39^. When co-expressed with NiV P, NiV N was maintained in a soluble state and was more efficient at impairing viral replication compared to when expressed alone, supporting the idea that the formation of nucleocapsid-like structures was not responsible for the restriction of viral replication.

Initiation of normal viral replicase activity for NiV, like other paramyxoviruses, is believed to occur after viral gene products have accumulated and the abundance of NiV N is suitable to interact with the viral polymerase mediating synthesis and encapsidation of the viral antigenome/genome^14,22–25,28,41^. Excess amounts of NiV N present at the time of infection could bind and encapsidate positive-sense viral RNA (not cellular RNA) as it is being synthesized, producing antigenome instead of mRNA and subsequently inhibit the production of viral proteins. While this is an intriguing hypothesis, our data demonstrating that a peptide can also impair viral replication would argue against this. It is improbable that the N- and C- terminal peptides are functionally able to encapsidate RNA, either viral or cellular. One study demonstrated that the minimum requirement of NiV N for nucleocapsid formation spans aa 30-404^44^. A subsequent study broke down the NiV N protein into 7 domains: the N-terminal arm (aa 1–45), the N-terminal domain-1 (aa 46–90), the N-terminal domain-2 (aa 91–157), the N-terminal domain-3 (aa 158–263), the C-terminal domain (aa 264–370), C-terminal arm (aa 371–385) and the C-terminal tail (aa 386–532)^39^. Of these domains only the N-terminal domain-3 and C-terminal domain appear to support RNA binding, of which the key residues required for binding RNA are found within NiV N at aa 178, 187, 192, 193, 352, and 354^39^. This study confirmed that RNA binding capacity is found within the central core of the protein; while the N- and C-terminal ends appear to be dispensable. Additionally, this has been supported by a previous study that shows that the C-terminal peptide, aa 468–532, is not required for RNA binding or nucleocapsid formation^44^ Furthermore, when expressed in cells, the peptides were diffusely distributed, suggesting a lack of nucleocapsid-like formation, unlike full-length NiV N which caused punctate staining (Supplemental Fig. 5). The ability of the two peptides, NiV N 1-55 and NiV N 468-532, to both bind NiV P, disrupt viral replication, and the lack of evidence for RNA binding suggests that RNA binding is not playing a role in the alteration of polymerase function.

The findings described in this study suggest the importance of maintaining routine interactions between NiV N and P for viral replication to occur. It has been previously suggested that N proteins interact directly with the polymerase complex to activate replicase activity in a manner that is independent of genome encapsidation^10,14,25,40^. Although this idea has not been fully developed and is the subject of much debate, it may provide an explanation for the results we observed in this study. If the N protein is able to initiate a change in polymerase function to replicase activity by a direct interaction with the polymerase complex via the P protein, we would expect viral RNA synthesis to be biased towards viral genome production, resulting in the accumulation of genomic RNA. We demonstrated a decline in both viral transcripts and genome production at 24 hpi. Similar findings have also been reported using a minigenome system to understand the replication strategy for respiratory syncytial virus, a paramyxovirus, and lymphocytic choriomeningitis virus, an arenavirus which has similar strategies for the production of viral RNA^11,45^. This finding is not unexpected as genome propagation would be impossible without the *de novo* production of viral proteins. However, when we assessed the replicase activity of the incoming polymerase after a single round of replication we were able to confirm that the increased presence of recombinant NiV N proteins led to an increase in replicase activity. This finding supports the proposal that polymerase function was skewed from transcriptase activity towards replicase activity by the direct interaction of the N protein with the P protein of the viral polymerase complex. Taking this one step further, the presence of NiV N peptides at the time of infection likely saturated the binding domain on the incoming NiV P protein of the polymerase complex, thereby disrupting transcriptase activity and/or inhibiting replicase activity; however, further studies would be needed to develop this idea more fully. Studies with influenza have also demonstrated that small-molecule compounds or viral peptides can cause structural interference between components of multimeric polymerase complexes, specifically the interaction between PA and PB1, and are capable of disrupting viral replication^46–48^.

Overall, to the best of our knowledge, this is the first time it has been demonstrated how altering the timing of NiV N expression has a negative impact on NiV RNA synthesis. While this is a new finding for NiV, these results also confirm previous reports for respiratory syncytial virus and lymphocytic choriomeningitis virus^11,45^. We also demonstrated that the preexisting recombinant NiV N protein was able to disrupt transcriptional activity of the incoming polymerase and instead led to the activation of its replicase activity. We identified that the interaction between NiV N and NiV P is a critical component to regulate viral RNA synthesis. The data presented here could provide an attractive avenue for development of potential antiviral prophylaxis or therapies by interfering with the structural interactions of the polymerase complex, mimicking what has been done in the influenza virus field^46–48^. The NiV replicase may be a suitable target for small-molecule inhibitors by interfering with the interactions between the N and P proteins viral replication can be impaired substantially.

## Materials and Methods

### Cells and Viruses

293 T and VeroE6 cells were cultured in Dulbecco’s modified Eagle’s medium (DMEM, Life Technologies, Burlington, ON, Canada) with 10% fetal bovine serum (FBS, Life Technologies, Burlington, ON, Canada). All cells were incubated in 5% CO_2_ and H_2_O-saturated atmospheric conditions at 37 °C. NiV-Malaysia (Accession No: NC_002728.1) was a kind gift from the Centers for Disease Control and Prevention (Atlanta, GA). Handling of NiV was done under containment level 4 (CL-4) conditions as outlined in the Health Canada Laboratory Bio-safety Guidelines CL-4 handling procedures (http://canadianbiosafetystandards.collaboration.gc.ca/cbs-ncb/index-eng.php). VSV serotype Indiana was kindly provided by Jack Rose from Yale University (New Haven, CT, USA)^49,50^.

### Cloning Strategy

The open reading frames (ORF) of NiV N and NiV P were amplified by RT-PCR from viral RNA. These genes were then cloned into the pBK-CMV expression vector (Agilent Technologies, Mississauga, Ontario, Canada). Sequential truncations were made to the NiV N gene in order to assess the various functional domains it may possess. Using Garnier-Osguthorpe-Robson prediction algorithms we designed our truncations in areas lacking any predicted secondary structure, in an attempt to minimize disruptions to the secondary structure and subsequent protein folding. The NiV N ORF was systematically truncated from the 3′ and/or the 5′ ends by PCR and a hemagglutinin (HA) tag was incorporated onto the 3′ end of the gene. These constructs were cloned into pBK-CMV. Larger truncations were designed and cloned into pEGFP-N1 creating a fusion protein which contains GFP on the C-terminal end of the protein. A dual promoter construct, NiV P-NiV N-CMV, was designed in order to express NiV N as well as NiV P from one construct. The dual promoter system was created by PCR amplifying the CMV promoter, the NiV N ORF, and the simian virus 40 (SV40) poly A termination signals and cloned downstream of the NiV P ORF in NiV P-CMV.

### Experimental Design

293 T cells were seeded onto 35 mm plates so that they were 70% confluent at the time of transfection. Increasing amounts of plasmid DNA was transfected into cells using Lipofectamine^TM^ 2000 (Life Technologies, Burlington, ON, Canada), as described by the manufacturer. Empty vector, either pBK-CMV or pEGFP-N1, was added to each sample to ensure equal amounts of plasmid DNA was transfected into cells. Transfection media was replaced 24 hours post-transfection with DMEM supplemented with 2% FBS, and the cells were incubated for another 24 hours. Cells were then infected with either NiV or VSV at an MOI of 1. Virus was adsorbed for 1 hour and then replaced with fresh media (DMEM/2% FBS). Cell lysates and supernatants were harvested 24 hours post-infection; cell lysates were collected for protein and RNA analysis, and supernatants were collected for protein and infectivity analysis.

### Northern Blots

Total RNA was isolated from cell lysates using TRIzol LS^®^ (Life Technologies, Burlington, ON, Canada) as recommended by the manufacturer. 5 μg of total RNA was separated by gel electrophoresis on a 1% Agarose – 7.4% formaldehyde gel and then transferred to a nylon membrane using a vacuum manifold. DNA probes designed against the NiV M gene and the GAPDH gene were labelled with α-dATP ^32^P (Redivue^®^ α-dATP ^32^P, GE Healthcare Life Sciences, Baie d’Urfe, Quebec, Canada) with the Random Primed Labelling Kit as per manufacturer’s instructions (Roche Life Sciences, Laval, Quebec, Canada). Blots were visualized using a Typhoon 9410 Variable Mode Imager and NiV M mRNA levels were standardized against GAPDH and quantified using ImageQuant 5.2 software.

### Western Blot

Cell lysates or supernatants were separated by SDS-PAGE and then transferred onto PVDF membranes using a semi-dry transfer apparatus. The membrane was blocked with 5% skim milk +0.1% Tween-20 and probed first with a primary antibody, followed by a secondary antibody conjugated to HRP diluted in blocking buffer. Nipah antibodies were developed in house, while antibodies against GFP were purchased from (Santa Cruz Biotechnology, Dallas, Texas, USA). Antibodies against VSV G and all secondary antibodies were purchased from (Sigma-Aldrich, Oakville, Ontario, Canada). The proteins of interest were visualized using the ECL + plus Western Blotting Detection System (GE Healthcare Life Sciences, Baie d’Urfe, Quebec, Canada). Blots were quantified using spot densitometry and AlphaEaseFC^TM^ software, and normalized against the expression of actin. Standard deviations of the mean were calculated.

### Strand-Specific Reverse Transcription and Real-Time PCR

Strand-specific reverse transcription (RT) and real-time PCR analyses were carried out using 1 µg of RNA extracted from total cell lysates using an RNeasy Plus Mini kit (Qiagen, Toronto, Ontario, Canada) that were harvested at 0, 6, 9 or 24 hpi. Primers and probes were designed from Nipah virus, strain Malaysia Assession No: NC_002728.1 (GenBank). RT reactions were carried out using a NiV L forward primer (NiV L fwd) or NiV L reverse primer (NiV L rev) to amplify either negative-sense NiV RNA or positive-sense NiV RNA, respectively, using High Capacity cDNA Reverse Transcription Kit (Thermo Fisher, Burlington, Ontario, Canada). Real-time PCR was then performed using the TaqMan(R) Fast Advanced Master Mix kit (Thermo Fisher, Burlington, Ontario, Canada) using both the NiV L fwd and rev primers along with a FAM labelled NiV L probe that targets a 65 base pair region of the NiV polymerase as per manufacturer’s instructions. Copy numbers were extrapolated from a standard curve using known concentrations of a DNA construct containing the entire viral genome of NiV.

NiV L fwd (bp 11939–11958): 5′-CAAAACAGAGATGCGAGCAG-3′

NiV L rev (bp 11985–12004): 5′-ATGCATGAATCTGAACGGAA-3′

NiV L probe (bp 19960–11984): 5′-FAM-GATCAAGAATTCRCAAAAGCCGAAA-BHQ1-3′

### Infectivity Assays

293 T cells were seeded into 24 well dishes 24 hours prior to infection so that they were 80% confluent at the time of infection. Supernatants from infected cells for titration were serially diluted and adsorbed onto cells for 1 hour. Inoculum was removed and replaced with fresh DMEM supplemented with 2% FBS. Cells were incubated for 48 hours. The presence of cell death and/or the formation of multi-nucleated giant cells (typical NiV-induced CPE) were analyzed and calculated into TCID_50_ infectious units (IFU) using the Spearman-Karber method^51^.

### Solubility Assay

Cells transfected with 4 μg of NiV N-CMV or NiV P-NiV N-CMV DNA were washed three times with sterile phosphate buffered saline (PBS) and lysed in PBS/0.1% Nonidet P40 (NP-40, Sigma-Aldrich, Oakville, Ontario, Canada) lysis buffer for 1 hour at 4 °C with end-over-end rotation. The cell lysate was clarified by centrifugation at 19,000 × g for 15 minutes and the supernatant was layered over 1.3 ml of a 20% sucrose cushion. The cushion was spun at 130,000 × g for 1 hour at 4 °C in a Beckman Optima^TM^ TLX Ultracentrifuge. The supernatant (soluble fraction) was collected and the pellet (insoluble fraction) was resuspended in an equal volume of sterile PBS. Sample analysis was done by western blot quantitation as previously described.

### Immunofluorescence Assay

293 T cells were seeded into 0.8 cm^2^-well chamber slides so that the cells were 60% confluent at the time of transfection. Cells were transfected with 0.3 μg of each plasmid using Lipofectamine^TM^ 2000, as described by the manufacturer, for 24 hours. Cells were fixed and permeabilized in 4.0% paraformaldehyde-PBS + 0.6% Triton^®^-X-100 (Sigma-Aldrich, Oakville, Ontario, Canada) and blocked in PBS supplemented with 1% Bovine Serum Albumin and 0.6% Triton^®^-X-100. A monoclonal antibody against NiV P generated in house was employed for the detection of cell-expressed NiV P, followed by the secondary antibody, goat anti-mouse-AlexaFluor^®^ 568 (Life Technologies, Burlington, ON, Canada), and finally, a monoclonal antibody generated in house, mouse anti-NiV N-FITC, against NiV N was added. An Olympus IX70 confocal microscope and Fluoview 2.1 software were used for acquisition of images. Cells were visualized at 60X magnification. Controls to ensure that cross-reactivity between antibodies or cells was not occurring were carried out for each experiment.

### Co-Immunoprecipitation Assays

293 T cells were seeded so that they were 70% confluent at the time of transfection. Cells were transfected with various NiV N constructs and NiV P-CMV. Transfected cells were lysed in PBS/0.1% NP-40 for 1 hour at 4 °C with end-over-end rotation. Lysates were cleared of cellular debris by centrifugation at 19,000 x g for 15 minutes. Cell lysates were pre-cleared by addition of the lysate to the Immobilized protein A/G resin (Thermo Fisher Scientific, Ottawa, Ontario, Canada) and incubated with end-over-end rotation. The pre-cleared lysate was removed by centrifugation and the lysate was then incubated with immobilized protein A/G resin (Thermo Fisher Scientific, Ottawa, Ontario, Canada) with conjugated monoclonal NiV P antibodies overnight at 4 °C. Proteins bound to the beads were eluted by boiling in 2X SDS sample buffer for 5 minutes. A reciprocal set of co-immunoprecipitations were employed to confirm any interactions using either an HA-Affinity Matrix (Roche Life Sciences, Laval, Quebec, Canada), or anti-GFP conjugated agarose (Santa Cruz Biotechnology, Dallas, Texas, USA), where truncated NiV N constructs were immunoprecipitated and NiV P was co-immunoprecipitated. Western blots were carried out to visualize the immunoprecipitated and co-immunoprecipitated proteins.

### Cell Viability Assays

293 T cells were transfected with NiV N-CMV as previously described. Forty-eight hours post-transfection, the media was removed and replaced with fresh OptiMEM (Life Technologies, Burlington, ON, Canada) supplemented with XTT (XTT-based InVitro Toxicology Assay Kit, Sigma-Aldrich, Oakville, Ontario, Canada) as per the manufacturer’s directions. The cells were incubated for a further 3 hours. The colorimetric change was read at an absorbance of 450 nm and percent viability was normalized to the control samples.

### Virus Assession Numbers

Nipah virus, strain Malaysia Assession No: NC_002728.1 (GenBank).

## Electronic supplementary material

Supplementary Information

## Data Availability

The datasets generated during and/or analysed during the current study are available from the corresponding author on reasonable request.
