# Supplementary material for: The interaction between the Nipah virus nucleocapsid protein and phosphoprotein regulates virus replication
Source: Sci Rep. 2018 Oct 30;8:15994. doi: 10.1038/s41598-018-34484-7 (PMC6207681; doi:10.1038/s41598-018-34484-7)

Supplemental Information

Title: The interaction between the Nipah virus nucleocapsid protein and phosphoprotein regulates virus replication

Running Title: Impairment of NiV Replication

Authors and Affiliation: Charlene Ranadheera[1,2#], Roxanne Proulx[2], Mark Chaiyakul[2], Shane Jones[2], Allen Grolla[2], Anders Leung[2],John Rutherford[1], Darwyn Kobasa[1,2], Michael Carpenter[1,3], Markus Czub[1,2,4]

[1]Department of Medical Microbiology, University of Manitoba, Winnipeg, Manitoba, Canada.

[2] Zoonotic Diseases and Special Pathogens, National Microbiology Laboratory, Public Health Agency of Canada, Winnipeg, Manitoba, Canada.

[3] Blood Borne Pathogens and Hepatitis, National Microbiology Laboratory, Public Health Agency of Canada, Winnipeg, Canada.

[4] Faculty of Veterinary Medicine, University of Calgary, Calgary, Alberta, Canada.

#Corresponding Author: charlene.ranadheera@canada.ca (CR)

Supplemental Figures

Supplemental Figure S1. Effects of recombinant NiV N expression on NiV replication in BHK cells. Cells were transfected with increasing amounts of plasmid DNA encoding the NiV N gene. 48 hours post transfection supernatants were harvested, viral loads were determined by endpoint titration and TCID50/ml was calculated. All experiments were carried out in triplicate. Standard deviations of the mean were calculated.

Supplemental Figure S2. Assessing the solubility of NiV N proteins in the presence or absence of NiV P proteins. (a) Lysates from cells expressing NiV N proteins alone or in conjunction with NiV P proteins were subjected to ultracentrifugation through a 20% sucrose cushion. The presence of NiV N in the soluble fraction (supernatant) or the insoluble fraction (pellet) was visualized by western blot using a monoclonal antibody against NiV N. Western blots were quantified by densitometry and normalized to the expression of actin. (b) Cells expressing NiV N proteins, NiV P proteins, or NiV N proteins in conjunction with NiV P proteins were analyzed by fluorescent confocal microscopy at a 60x magnification employing a Z-axis slice. A monoclonal antibody against NiV N conjugated with a FITC fluorochrome were used to detect NiV N proteins and are represented by green. A monoclonal antibody against NiV P used in conjunction with a secondary antibody containing an AlexaFluor® 568 fluorochrome were used to detect NiV P proteins and are represented by red. Co-localization between NiV N and NiV P proteins is represented by yellow.

Supplemental Figure S3. Effects of increasing amounts of recombinant NiV P protein co-expressed with constant levels of recombinant NiV N protein on viral replication. Cells were transfected with increasing amounts of plasmid DNA encoding the NiV P gene and 4 µg of a plasmid encoding the NiV N gene for 48 hours. Following transfection, (a) a western blot was performed using cell lysates to confirm the increasing expression of NiV P protein and the constant expression of NiV N in cells. (b) A parallel set of cells were infected with NiV at an MOI of 1 for 24 hours. Supernatants were harvested for titration of viral loads. All experiments were done in triplicate and standard deviations of the mean were calculated.

Supplemental Figure S4. Effects of co-expression of recombinant NiV P with NiV N 1-54-GFP or NiV N 468-532-GFP on NiV replication. Cells were transfected with increasing amounts of plasmid DNA encoding (a) NiV N 1-54-GFP or (b) NiV N 468-532-GFP gene and constant amounts of the NiV P gene. 48 hours post transfection supernatants were harvested, viral loads were determined by endpoint titration and TCID50/ml was calculated. All experiments were carried out in triplicate. Standard deviations of the mean were calculated.

Supplemental Figure S5. Expression Patterns of NiV-GFP Constructs. Cells were transfected with DNA plasmids expressing NiV N-GFP, NiV N 1-54-GFP, and NiV N 468-532-GFP. 24 hours post transfection cells were analyzed by fluorescent microscopy at a 20x magnification.

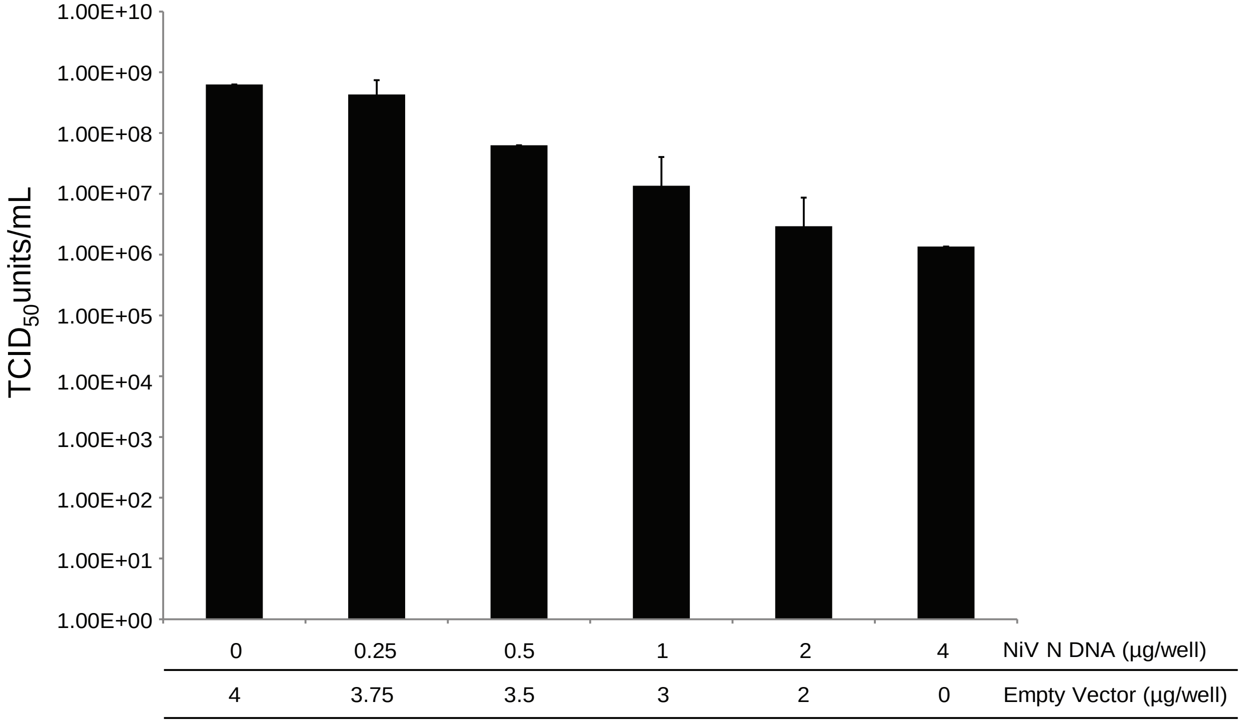

Supplemental Figure S1. Effects of recombinant NiV N expression on NiV replication in BHK cells. Cells were transfected with increasing amounts of plasmid DNA encoding the NiV N gene. 48 hours post transfection supernatants were harvested, viral loads were determined by endpoint titration and TCID50/ml was calculated. All experiments were carried out in triplicate. Standard deviations of the mean were calculated.

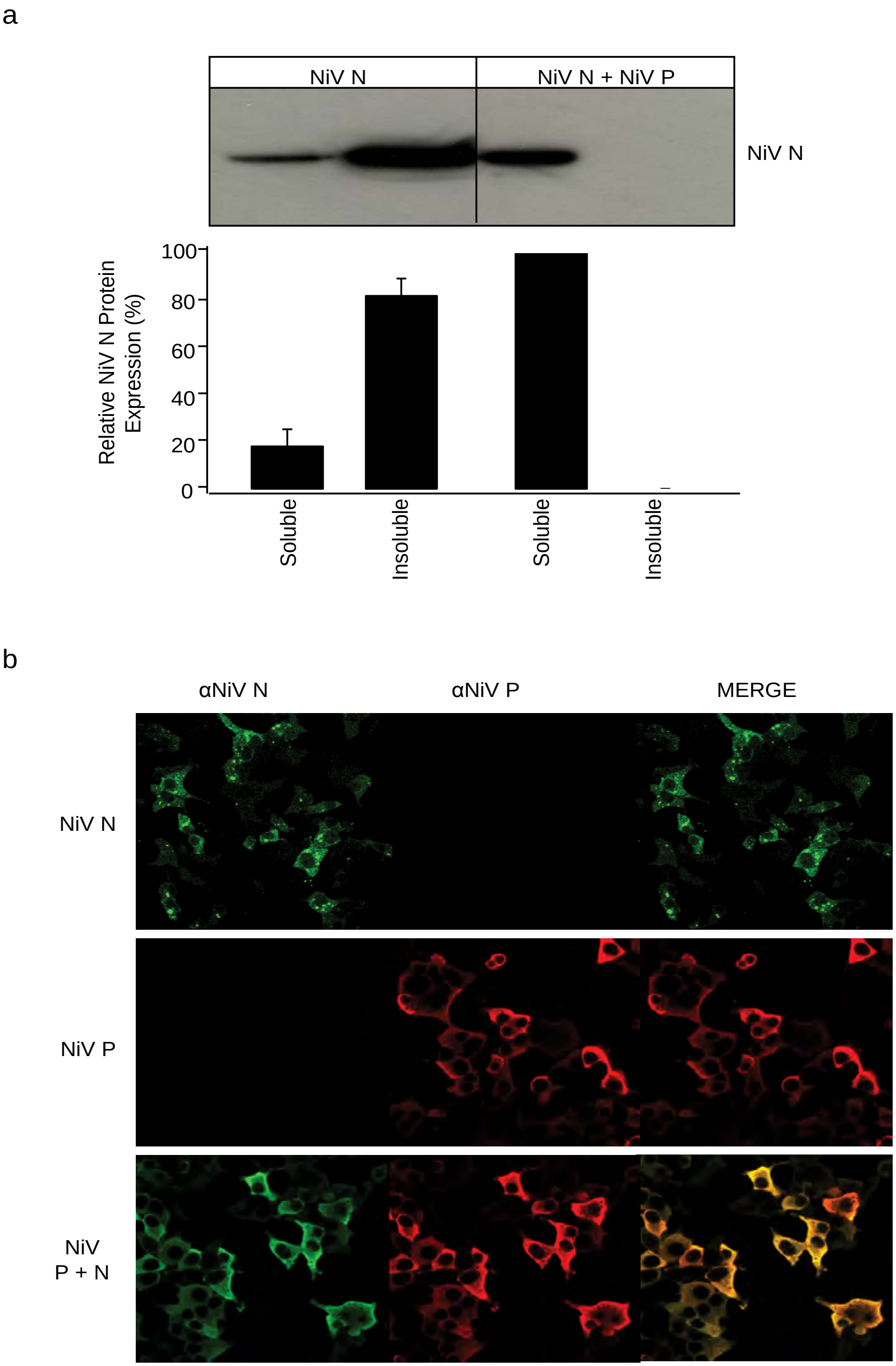

Figure S2. Assessing the solubility of NiV N proteins in the presence or absence of NiV P proteins. (a) Lysates from cells expressing NiV N proteins alone or in conjunction with NiV P proteins were subjected to ultracentrifugation through a 20% sucrose cushion. The presence of NiV N in the soluble fraction (supernatant) or the insoluble fraction (pellet) was visualized by western blot using a monoclonal antibody against NiV N. Western blots were quantified by densitometry and normalized to the expression of actin. (b) Cells expressing NiV N proteins, NiV P proteins or NiV N proteins in conjunction with NiV P proteins were analyzed by fluorescent confocal microscopy at a 60x magnification employing a Z-axis slice. A monoclonal antibody against NiV N conjugated with a FITC fluorochrome were used to detect NiV N proteins and are represented by green. A monoclonal antibody against NiV P used in conjunction with a secondary antibody containing an AlexaFluor®568 fluorochrome were used to detect NiV P proteins and are represented by red. Co-localization between NiV N and NiV P proteins is represented by yellow.

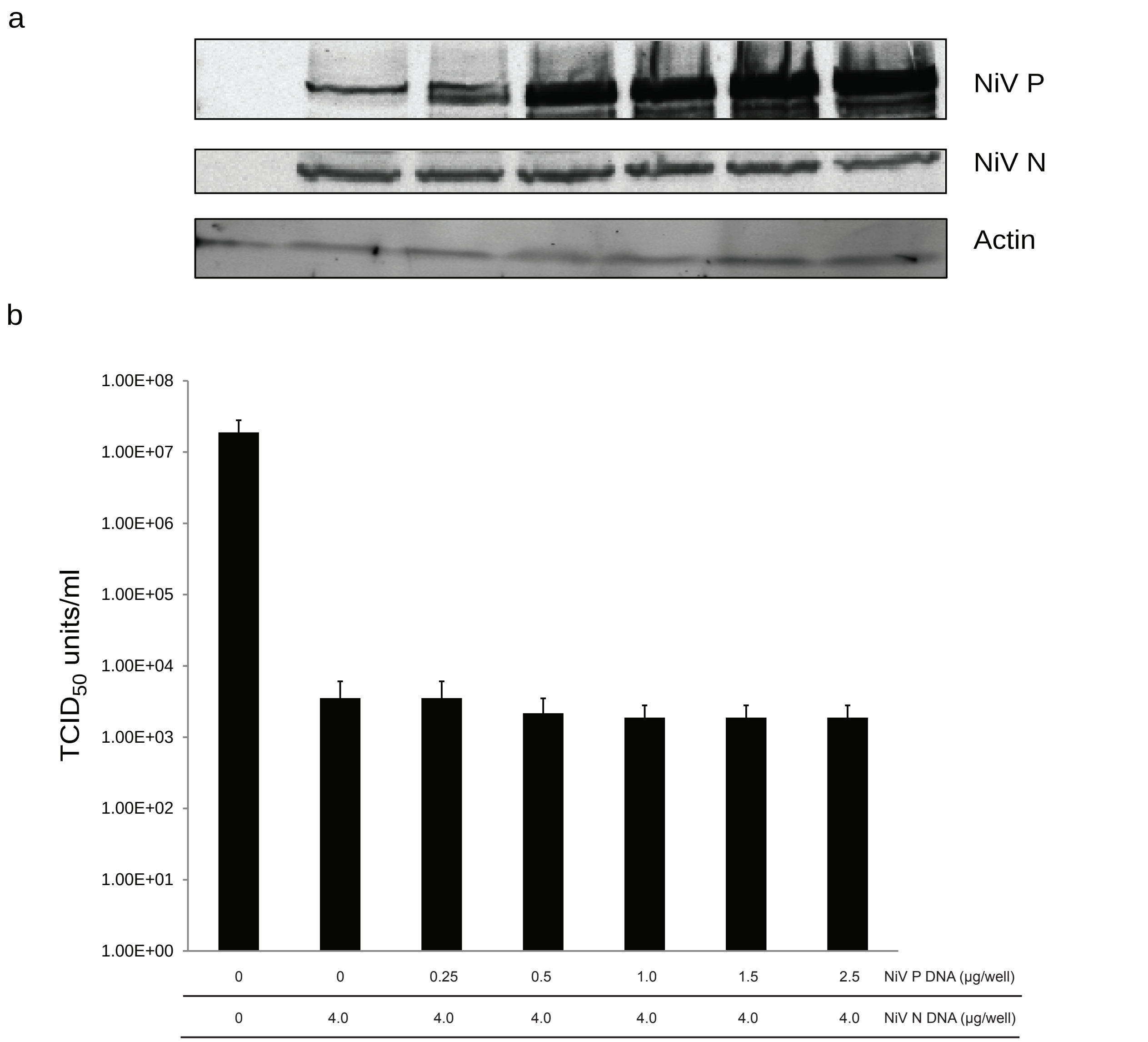

Figure S3. Effects of increasing amounts of recombinant NiV P protein co-expressed with constant levels of recombinant NiV N protein on viral replication. Cells were transfected with increasing amounts of plasmid DNA encoding the NiV P gene and 4 µg of a plasmid encoding the NiV N gene for 48 hours. Following transfection (a) a western blot was performed using cell lysates to confirm the increasing expression of NiV P protein and the constant expression of NiV N in cells. (b) A parallel set of cells were infected with NiV at an MOI of 1 for 24 hours. Supernatants were harvested for titration of viral loads. All experiments were done in triplicate and standard deviations of the mean were calculated.

a

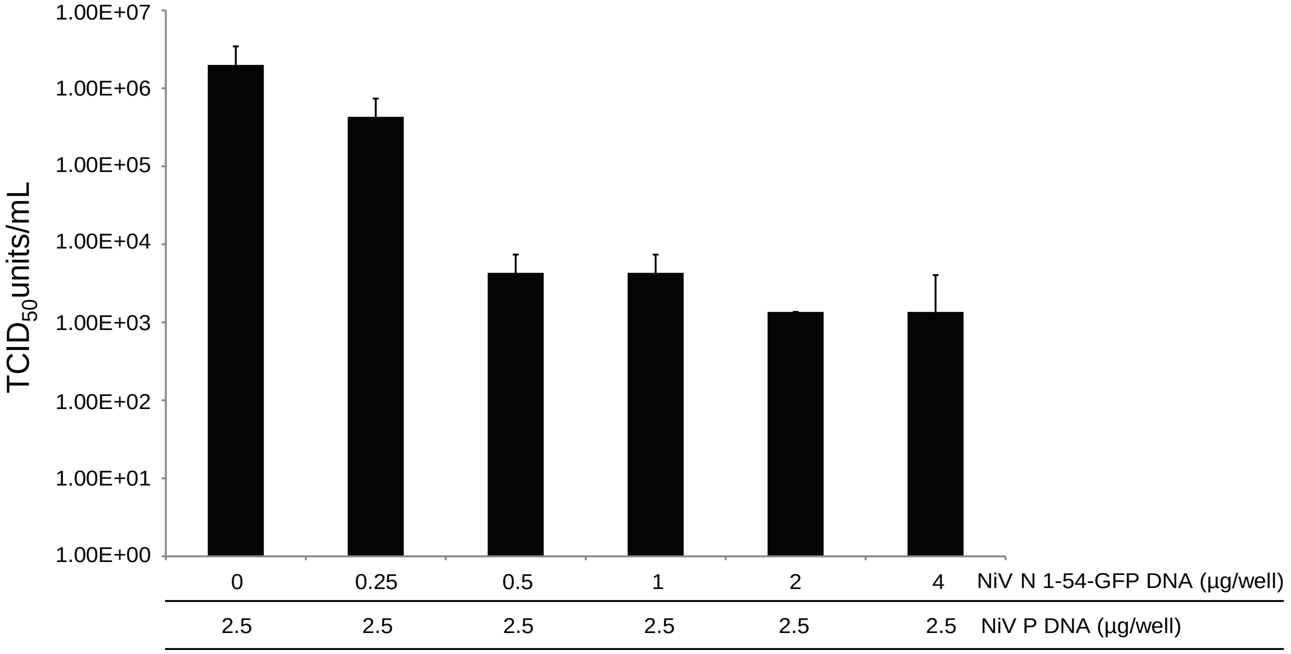

b

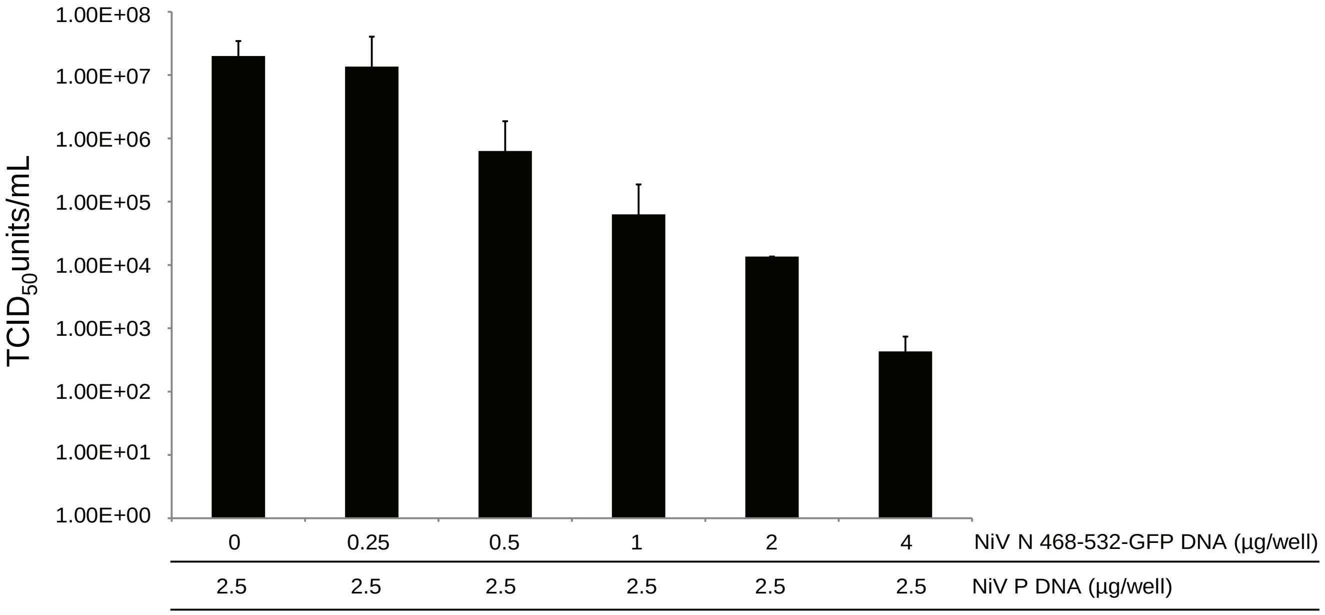

Supplemental Figure S4. Effects of co-expression of recombinant NiV P with NiV N 1-54-GFP or NiV N 468-532-GFP on NiV replication. Cells were transfected with increasing amounts of plasmid DNA encoding (a) NiV N 1-54-GFP or (b) NiV N 468-532-GFP gene and constant amounts of the NiV P gene. 48 hours post transfection supernatants were harvested, viral loads were determined by endpoint titration and TCID50/ml was calculated. All experiments were carried out in triplicate. Standard deviations of the mean were calculated.

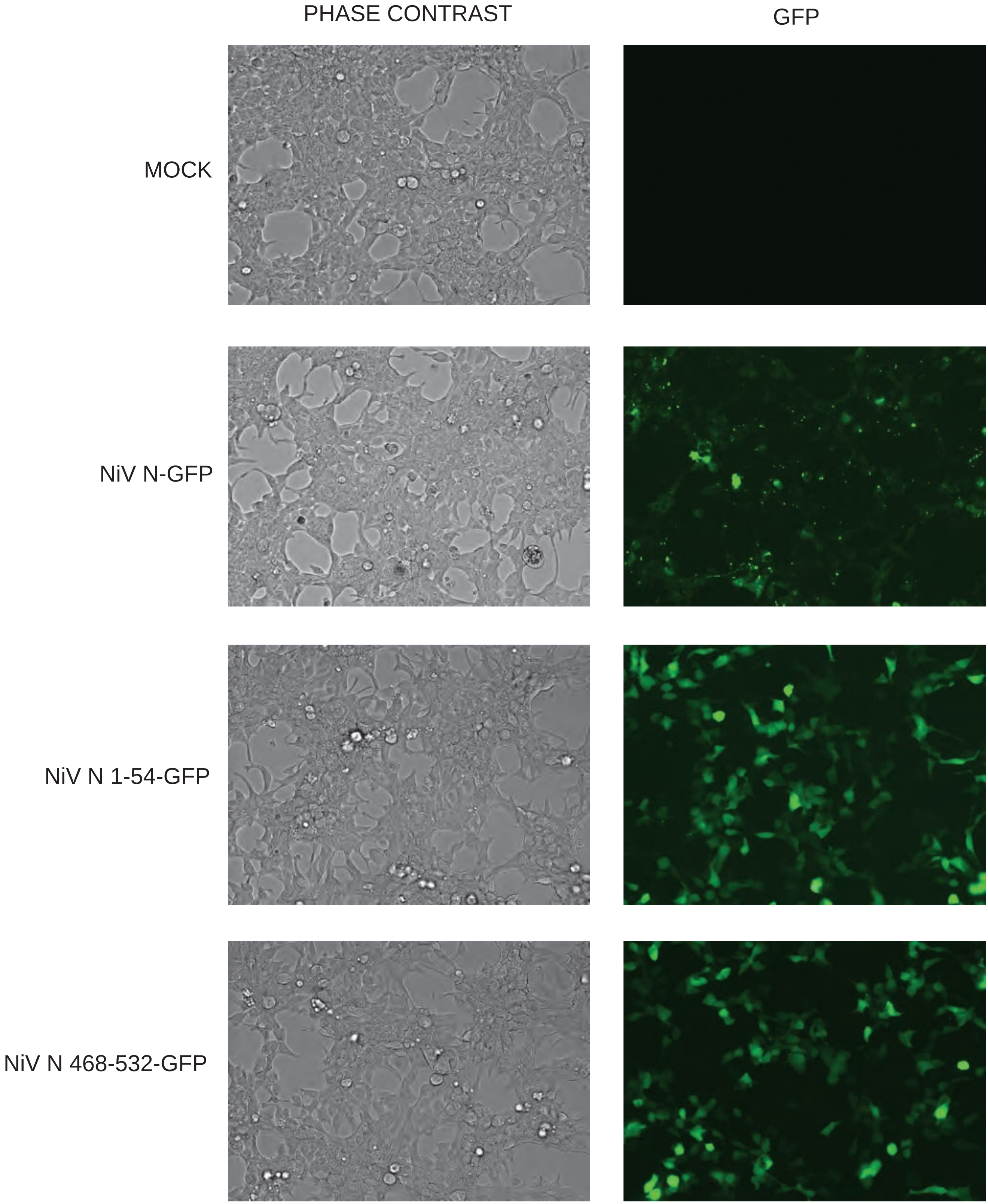

Supplemental Figure 5. Expression Patterns of NiV-GFP Constructs. Cells were transfected with DNA plasmids expressing NiV N-GFP, NiV N 1-54-GFP, and NiV N 468-532-GFP. 24 hours post transfection cells were analyzed by fluorescent microscopy at a 20x magnification.

Supplemental Information:

Full Size Western Blot Images as shown in the manuscript

Figure 1a:
Detection of NiV N and Actin

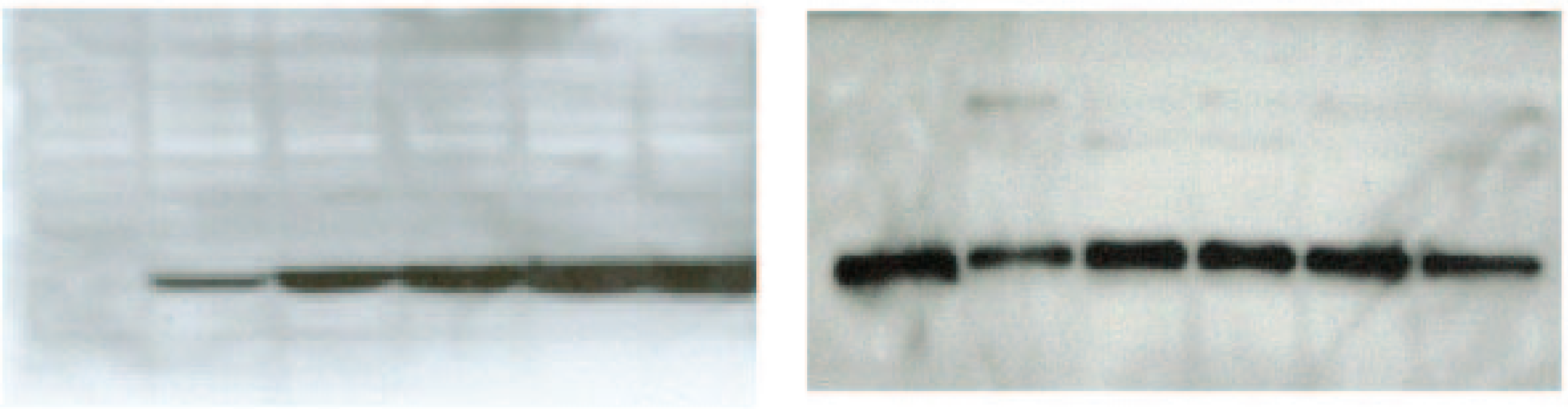

Figure 1b:
Detection of NiV M mRNA and GAPDH mRNA

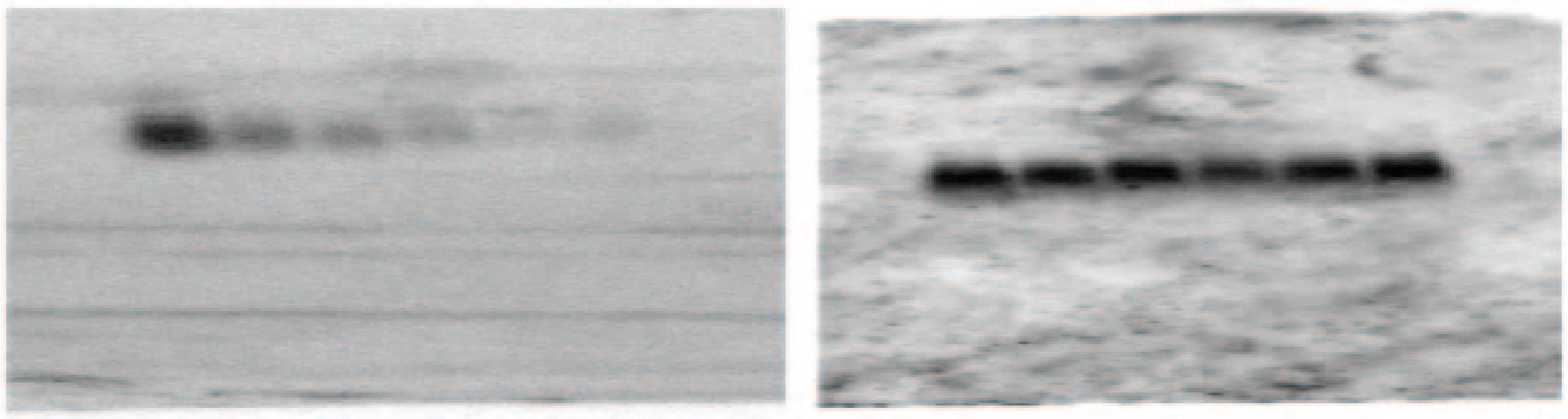

Figure 1c:
Detection of NiV P and Actin

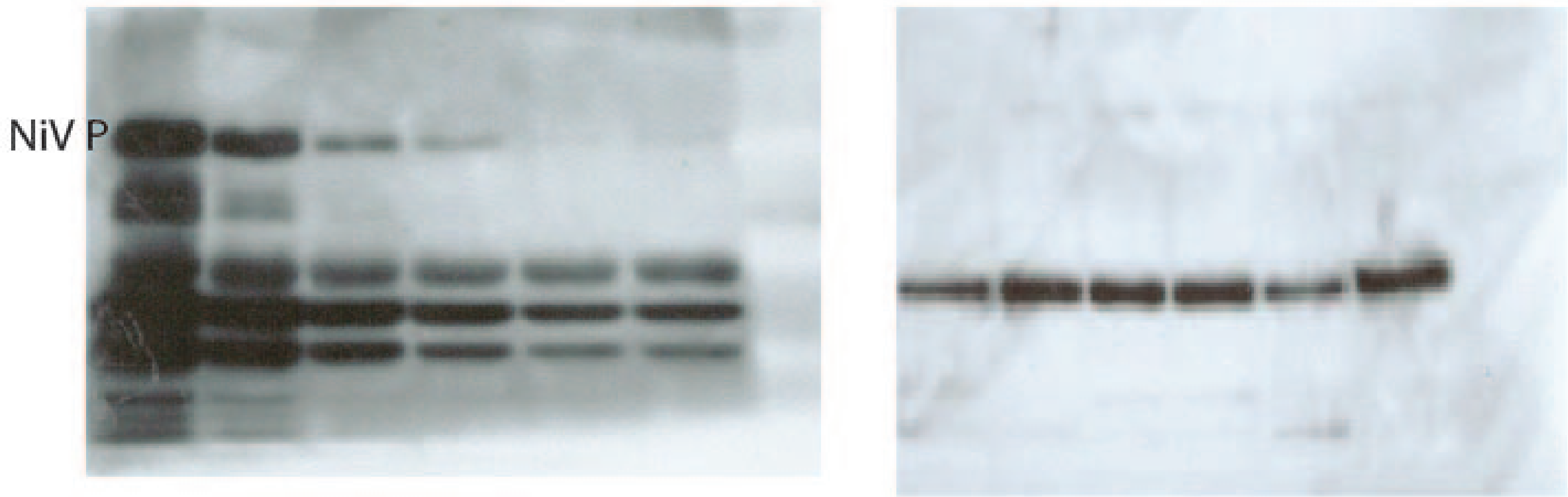

Figure 2a:
Detection of NiV P and Actin

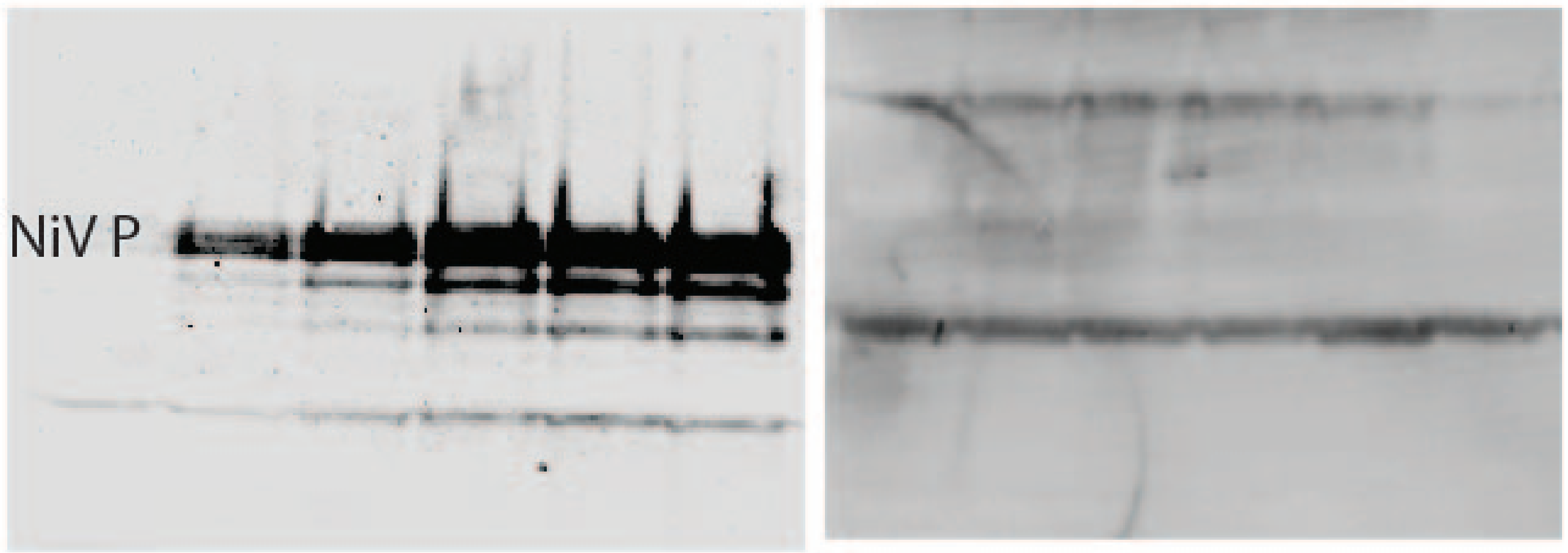

Figure 2b:
Detection of NiV N and Actin

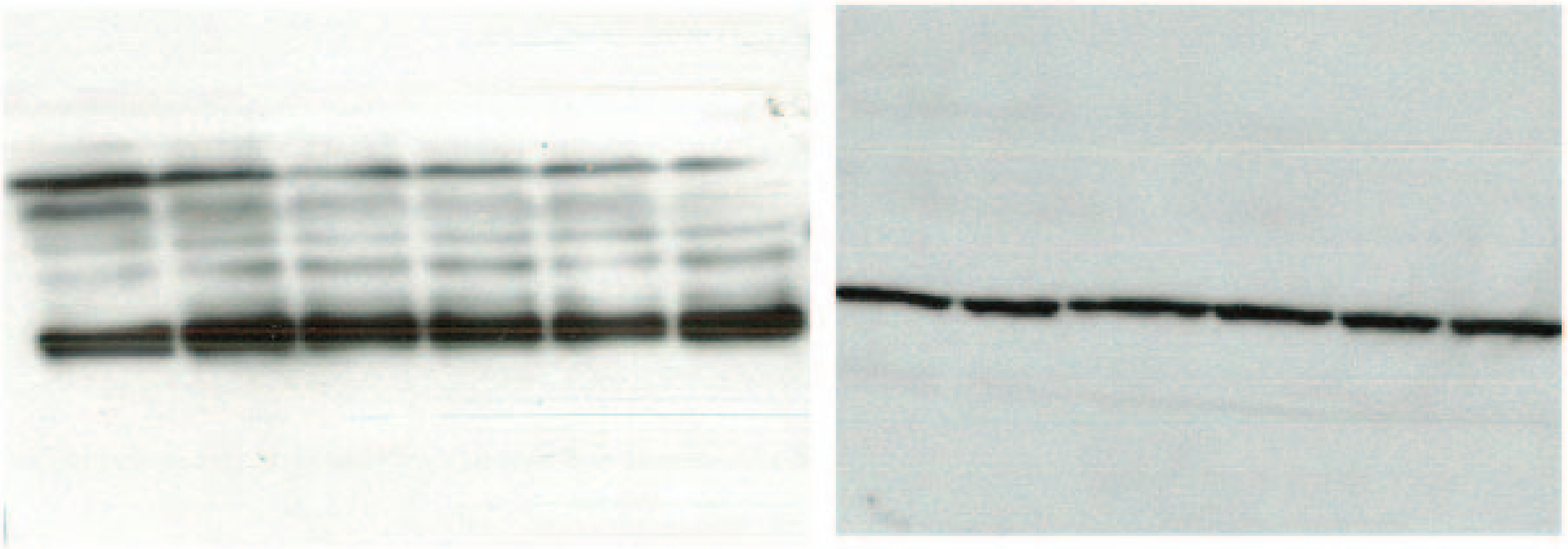

Figure 2c:
Detection of NiV F1 and Actin

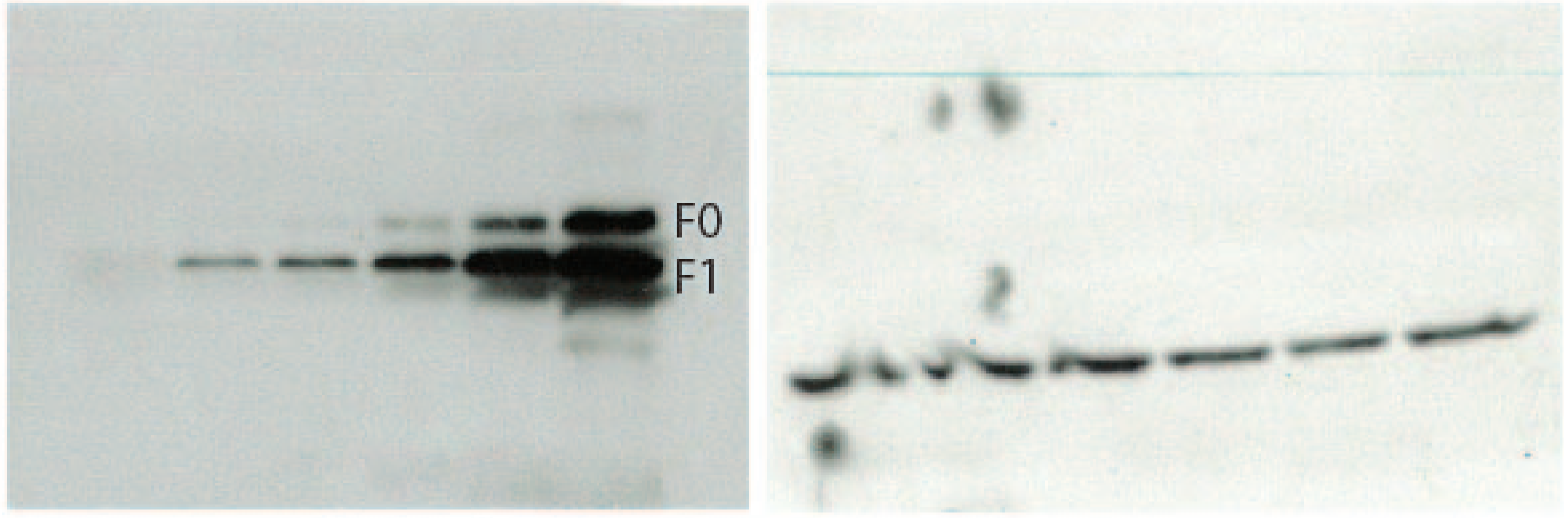

Figure 2d:
Detection of NiV P and Actin

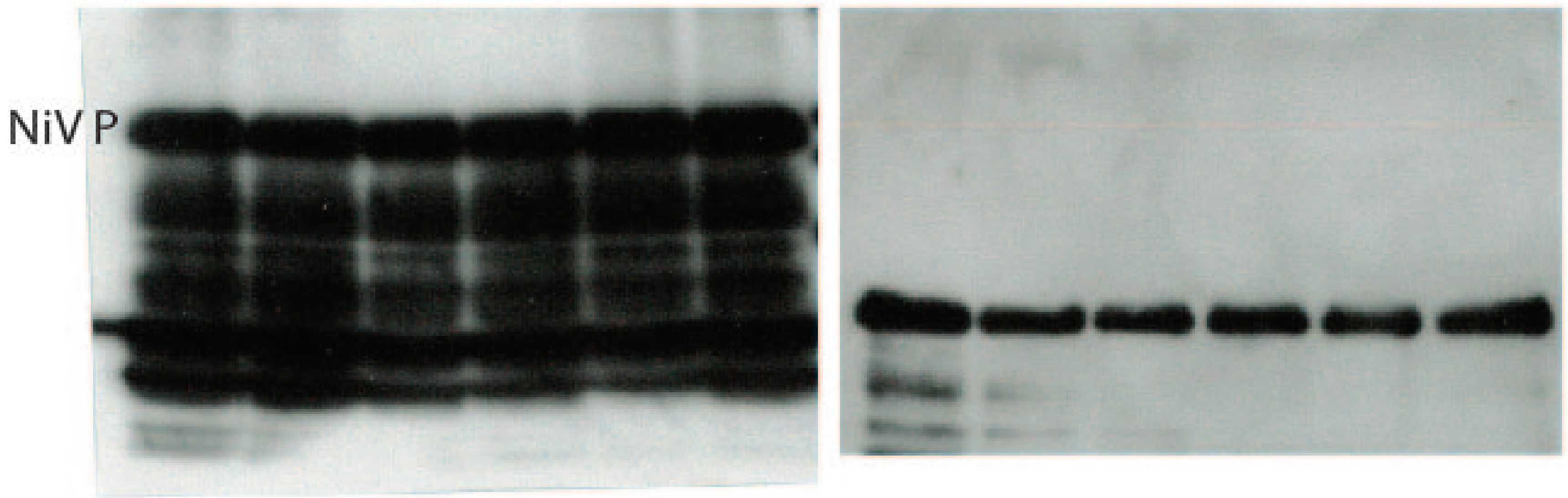

Figure 2e:
Detection of GFP and Actin

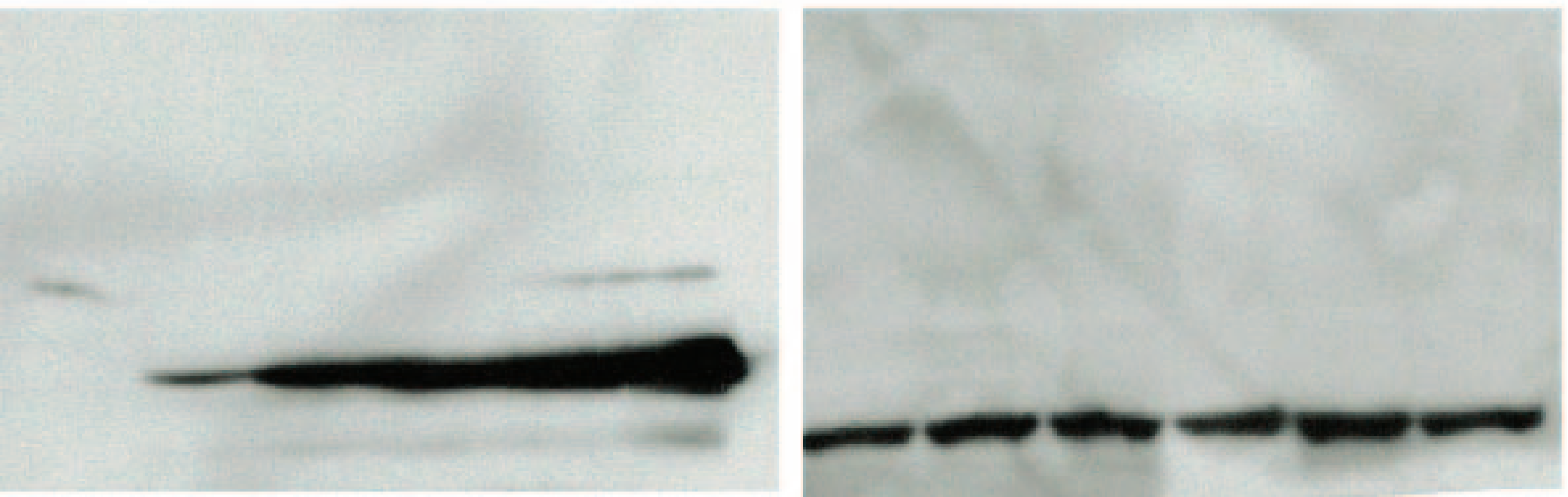

Figure 2f:
Detection of NiV P and Actin

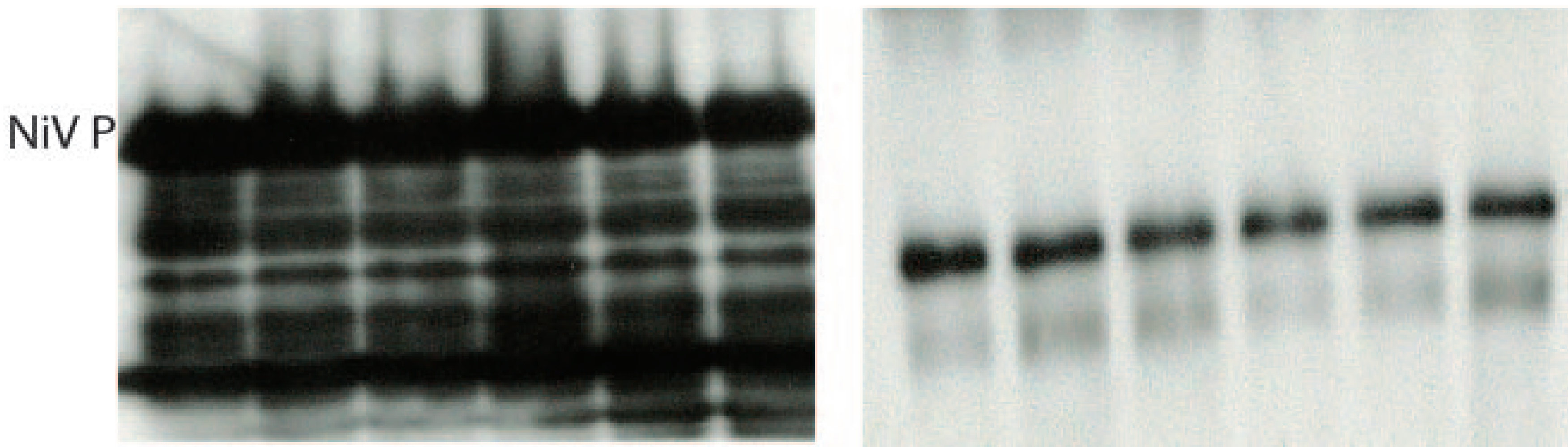

Figure 3a:
Detection of NiV N mRNA, GAPDH mRNA, NiV N protein, Actin protein

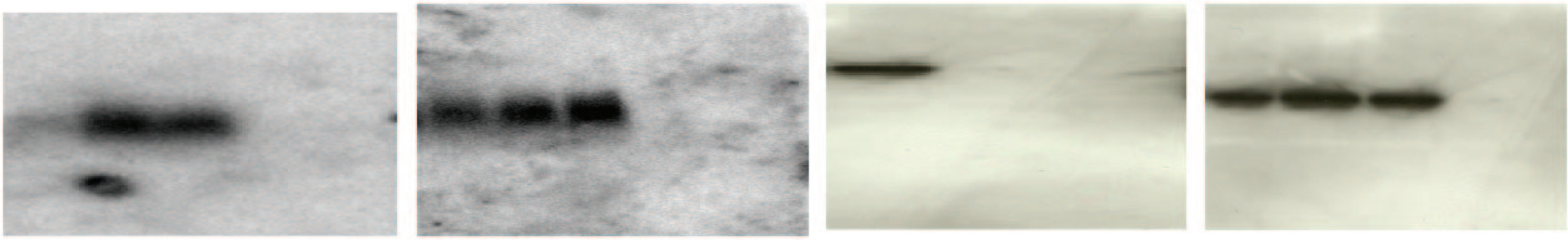

Figure 3b:
Detection of NiV P protein and Actin

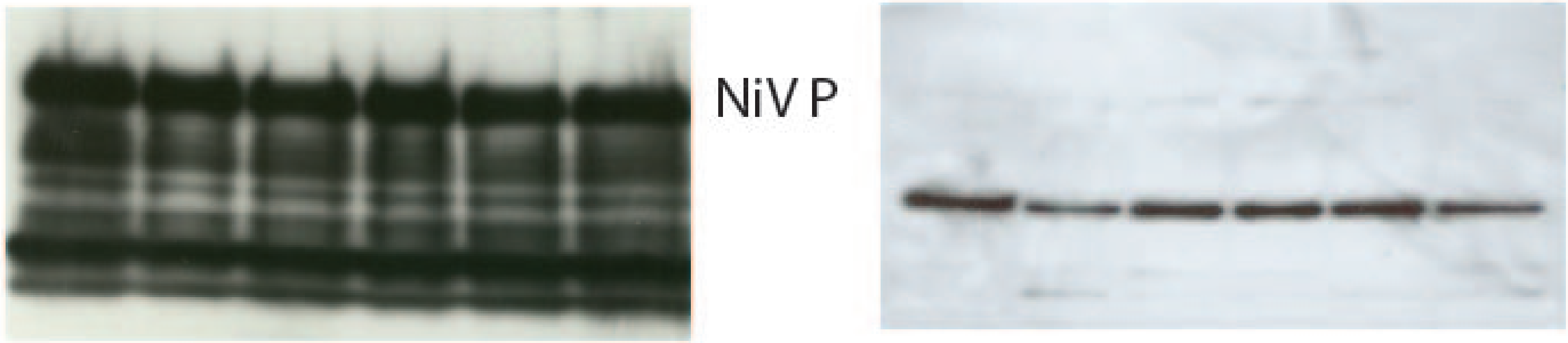

Figure 4b:
Detection of VSV G and Actin

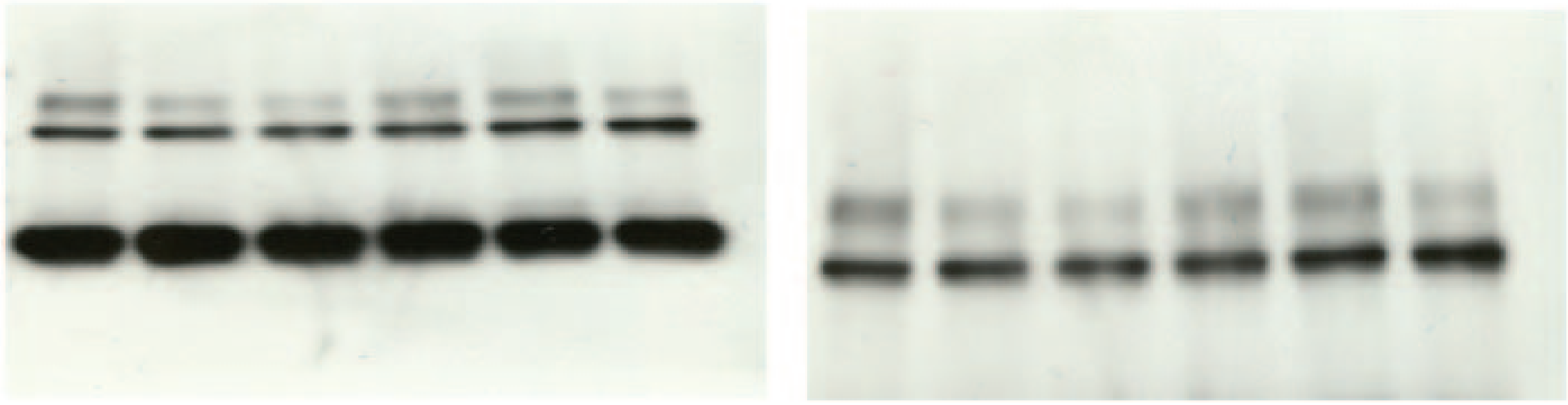

Figure 5a:
Detection of NiV N, NiV P, and Actin

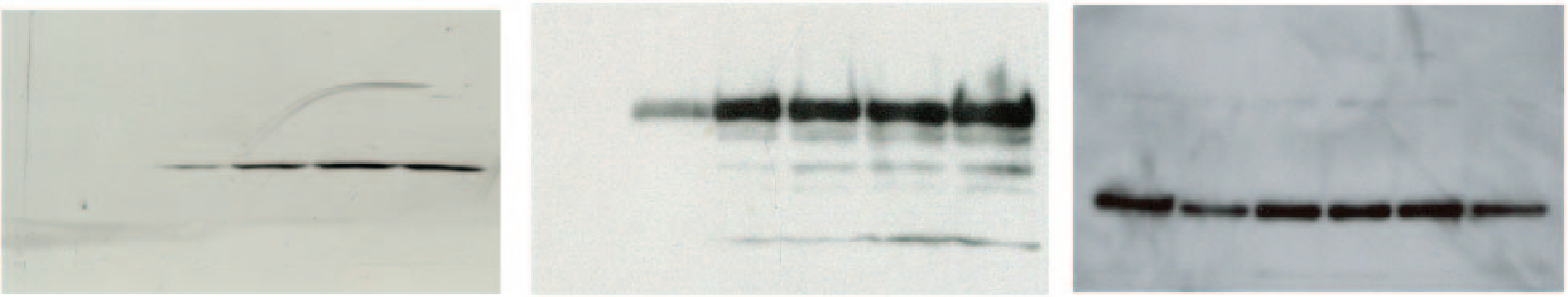

Figure 5b:
Detection of NiV M mRNA and GAPDH mRNA

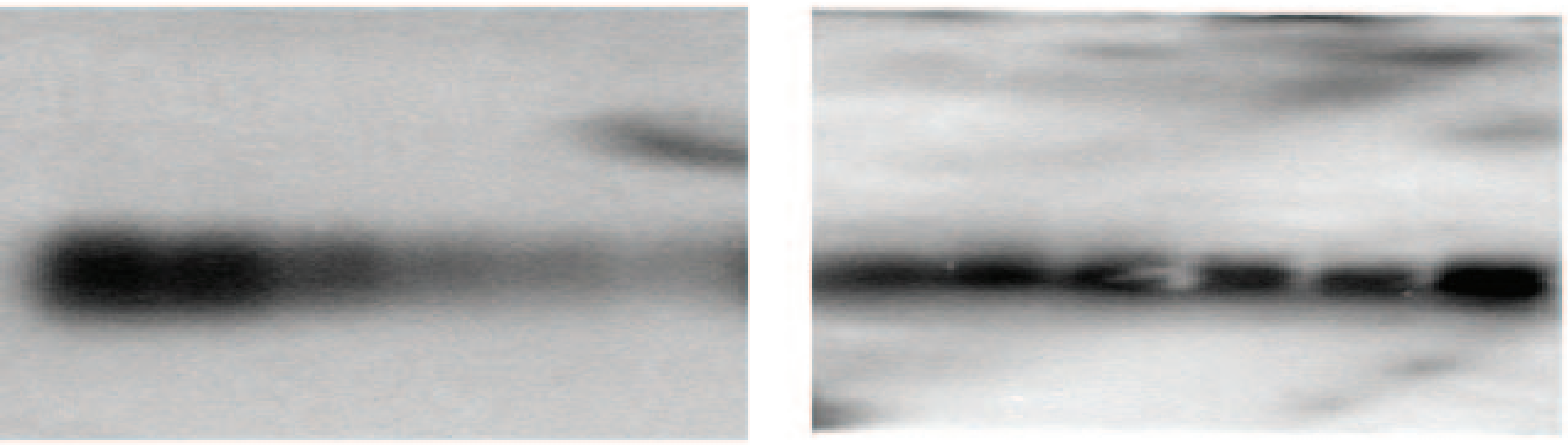

Figure 7a:
Detection of truncated NiV N total cell lysate, truncated NiV N coimmunoprecipitated, and NiV P immunoprecipitated

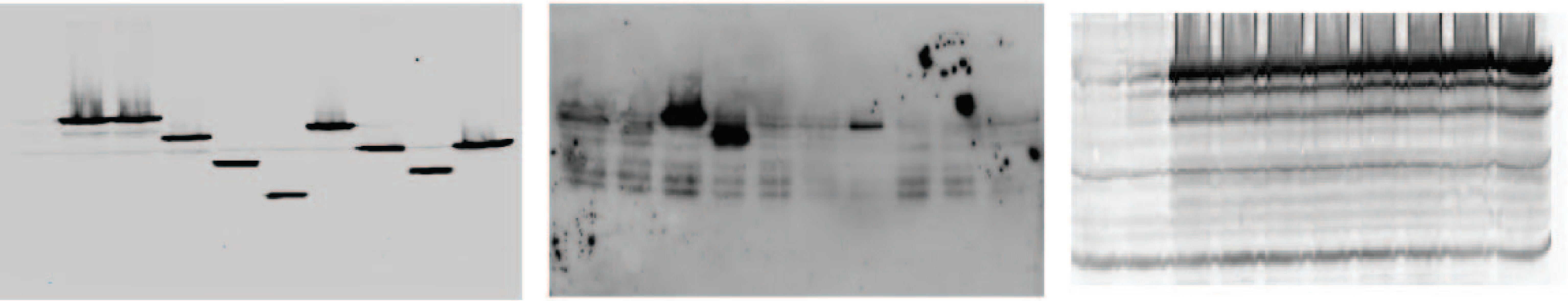

Figure 7b:
Detection of truncated NiV N total cell lysate, truncated NiV N coimmunoprecipitated, and NiV P immunoprecipitated

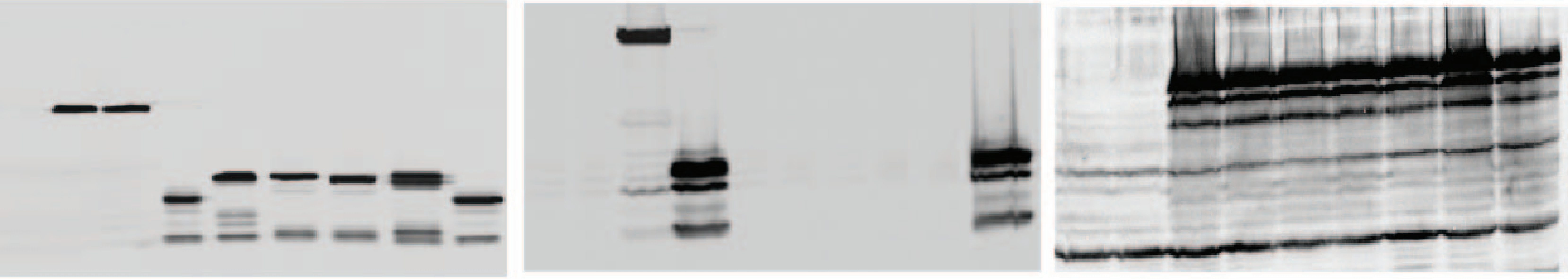

Supplemental Figure S2:
Detection of NiV N and P, and Actin

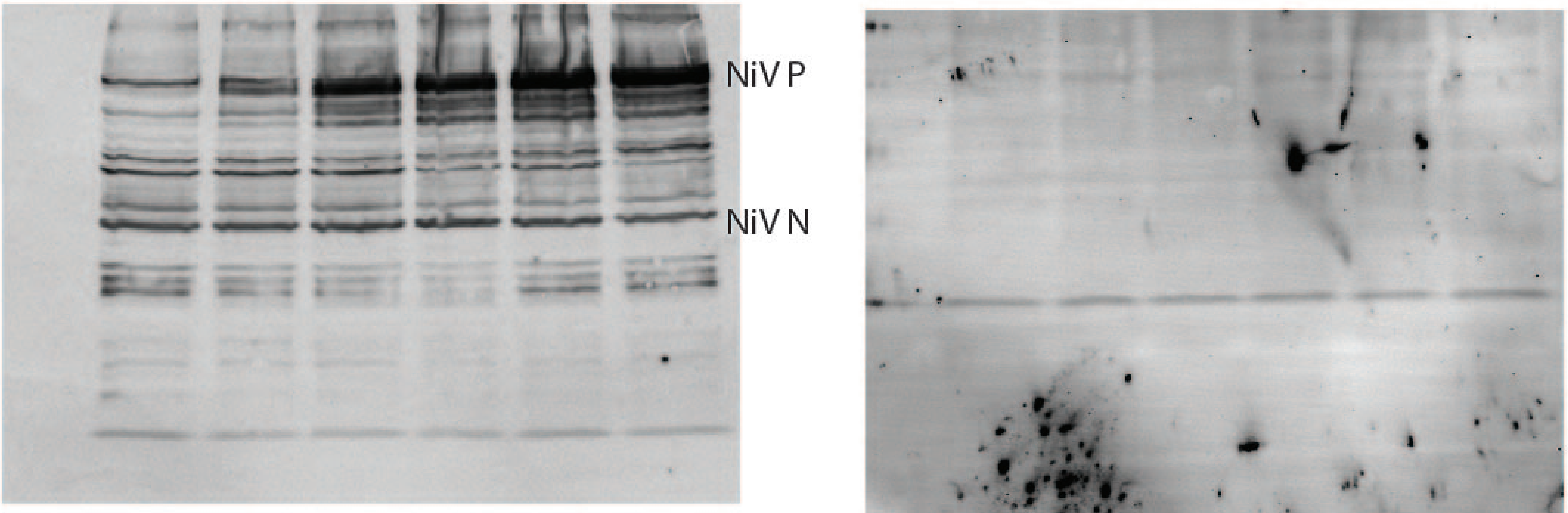

Supplement: Supplementary file 1 — Supplementary Information [file 41598_2018_34484_MOESM1_ESM.pdf]
